# Recent Developments in Pharmaceutical Spray Drying: Modeling, Process Optimization, and Emerging Trends with Machine Learning

**DOI:** 10.3390/pharmaceutics17121605

**Published:** 2025-12-13

**Authors:** Waasif Wahab, Raya Alshamsi, Bouta Alharsousi, Manar Alnuaimi, Zaina Alhammadi, Belal Al-Zaitone

**Affiliations:** Department of Chemical and Petroleum Engineering, United Arab Emirates University, Sheikh Khalifa Bin Zayed Street, Al-Ain 15551, United Arab Emirates

**Keywords:** spray drying, drug delivery, CFD, single droplet modeling, machine learning, hybrid ML models, digital twin, transfer learning, FDA/EMA guidelines, XAI

## Abstract

Spray drying techniques are widely used in the pharmaceutical industry to produce fine drug powders with different properties depending on the route of administration. Process parameters play a vital role in the critical quality attributes of the final product. This review highlights the progress and challenges in modeling the spray-drying process, with a focus on pharmaceutical applications. Computational fluid dynamics (CFD) is a well-known method used for the modeling and numerical simulation of spray drying processes. However, owing to their limitations, including high computational costs, experimental validation, and limited accuracy under complex spray drying conditions. Machine learning (ML) models have recently emerged as integral tools for modeling/optimizing the spray drying process. Despite promising accuracy, ML models depend on high-quality data and may fail to predict the influence of new formulation or process parameters on the properties of the dried powder. This review outlines the shortcomings of CFD modeling in the spray drying process. A hybrid model combining ML and CFD models, emerging techniques such as the digital twin approach, transfer learning, and explainable AI (XAI) are also discussed. A hybrid model combining ML and CFD models is also discussed. ML is considered an emerging technique that could assist the spray drying process, and most importantly, the utilization of this method in pharmaceutical spray drying.

## 1. Introduction

Spray drying is an energy-intensive, fast, continuous, and highly efficient method for producing solid particles from liquid solutions, suspensions, emulsions, slurries, pastes, and melts [1]. This process can produce nano- to micron-sized particles in a short time, ca. 2–4 s. Spray drying has various applications in the food, chemical, biochemical, and pharmaceutical industries [2]. Spray drying is frequently used in the food industry to produce various food products, such as milk powder, cheese powder, egg powder, juice powder, instant coffee/tea powder, color, and flavoring ingredients [3]. Fruit and vegetable powders produced by spray drying are more stable than liquid juice and can reduce the costs associated with packaging, storage, and transportation [4]. Moreover, spray-dried fruit and vegetable powders can be preserved for a longer period, depending on the packaging, compared to their liquid form [5]. Spray drying is widely used to encapsulate essential oils [6] and probiotic powders in dairy products [7]. The spray drying market is huge and growing worldwide, with market share estimated to reach between $6–$7 billion by 2025 [8]. The food industry is the biggest market for spray drying equipment, with a compound annual growth rate (CAGR) varying from 5.7% to 6.8% between 2019 and 2025 [8,9] The pharmaceutical sector is responsible for over 80% of the market’s value in terms of products such as active pharmaceutical ingredients (APIs) and excipients. It is expected to achieve a steady 17% growth rate until 2028 [10].

The spray drying process has various benefits, such as ease of operation, energy efficiency, and low operational costs; control over particle size, shape, and morphology; high encapsulation efficiency and product stability; and the ability to be scaled to the industrial level [11]. Some of the other currently available methods for drying of products include freeze drying, fluidized bed drying, spray–freeze drying, electro spraying, solar drying, superheated-steam drying, infrared drying, and supercritical fluid drying. Although these methods have been widely used for drying particles in various applications, such as food, pharmaceuticals, and chemicals, there are many limitations to these techniques, as summarized in Table 1.

In the pharmaceutical industry, spray drying is frequently used to alter the size distribution, crystallinity, morphology, and moisture content of spray-dried particles and to improve powder compactability [43,44,45]. Dry particles with narrow and respirable size distributions of 1–5 µm can be formulated using a spray drying process for effective pulmonary drug delivery [46]. Most modern therapeutic drugs show poor aqueous solubility and low drug dissolution rates, which reduce their bioavailability and slow the absorption process. Spray drying can produce a stable amorphous dispersion containing an active pharmaceutical ingredient (API) and polymer, thereby improving the drug dissolution rate [47]. Engineered particles produced by spray drying showed consistent dosing and deep lung deposition [48]. Spray drying permits the drying of heat-sensitive materials such as proteins or enzymes without losing their pharmacological benefits [49].

**Figure 1:**
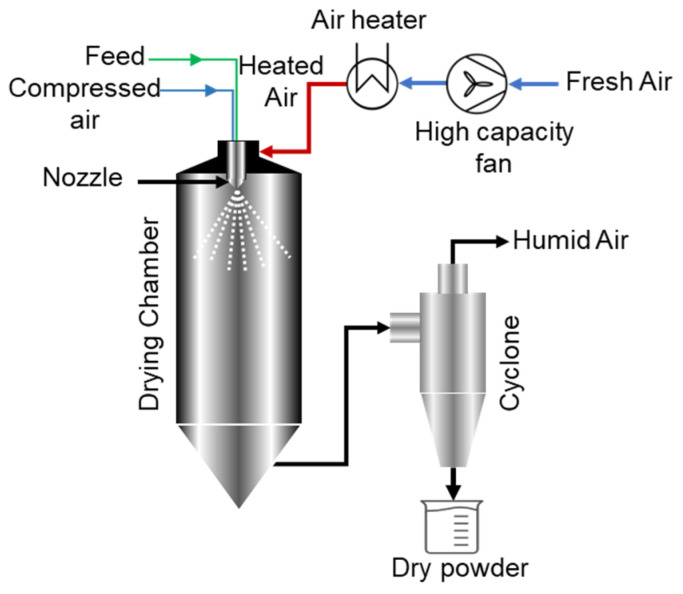
Spray drying process flow diagram, reproduced from [51] with permission from Springer Nature, 2025.

As illustrated in Figure 1, the spray drying process begins with the liquid feed solution being fed into the drying chamber along with hot gas, which atomizes the feed solution into fine droplets. The drying of droplets into fine powder occurs immediately as the hot drying gas simultaneously enters the drying chamber, either as a co-current or counter-current flow, and encounters the droplets [50]. The dried powder is then separated from the drying gas using a cyclone separator and collected in a collection vessel.

Spray-dried API powder generally consists of an excipient or carrier that acts as a binder, sweetener, or coating agent, or improves the performance of the API for different routes of drug delivery. Excipients such as cyclodextrin, poloxamers [50], and polydimethylsiloxanes [52] have been widely used for oral, nasal, and transdermal drug delivery. Drug particles less than 5 µm in size stick to larger carrier particles such as lactose (60–90 µm) through physical forces such as van der Waals, electrostatic, capillary, and mechanical interlocking forces that are vital for drug detachment and lung deposition during inhalation. The strength of these forces directly influences the efficiency of drug delivery from DPIs. Stronger forces can decrease dispersion and lung deposition [53] Molecular-level incompatibilities can happen between drug and an excipient, which can create crystal strain, influencing the uniformity and flowability of the powder mixture. The Maillard reaction is a specific chemical incompatibility that can result in dose reduction or toxic byproducts in DPI formulations [54]. Surface modification of excipients with magnesium stearate or Aerosil R972 can reduce these interactions, leading to improved bulk properties and DPI performance [55]. Spray-dried dry powders are generally delivered via dry powder inhalers (DPIs). Excipients such as lactose monohydrate have been used as coarse carriers in low-dose DPIs because of their nontoxicity, stability, and compatibility with most active drugs [56,57]. In high-dose DPIs, coarse carriers decrease the final powder volume by acting as a bulking agent. The major challenge of the micronized drug particles in high-dose DPIs is poor flowability and cohesion, resulting in particle agglomeration and incorrect dosing [58]. The addition of coarse carriers to the drug increases the bulk density, thereby improving the flowability of powder and reducing the dosage requirement compared to the drug alone [59]. Trehalose is another excipient that has been used as a carrier in DPI formulations, such as albuterol sulfate, disodium cromoglycate, and fluticasone propionate [60]. Amino acids such as L-leucine and tri-leucine are excipients that can improve the aerosol performance of spray-dried DPIs [61] and protect spray-dried inhalable powders from moisture [62]. D-mannitol is widely used as an excipient in pharmaceutical drugs because of its ability to absorb moisture, chemically inactive nature, and non-decomposable and non-degradable tendencies in reaction with drugs [63].

Nasal powders containing spray-dried excipients and drugs have demonstrated effective intranasal drug delivery in rabbits [64]. Bioavailability is one of the main concerns in oral drug delivery. Carrier particles, when spray-dried along with a poorly water-soluble drug, significantly improved oral bioavailability and drug efficiency in rats and mice compared to the pure drug [65]. Drug dissolution rate and fine particle fraction are vital for the bioavailability and therapeutic effectiveness of drugs. Carrier microparticles containing drug nanoparticles prepared by spray drying showed improved drug dissolution rates and higher fine-particle fractions for oral delivery [66]. For transdermal drug delivery, the spray-dried excipient drug powder decreased toxicity and improved the efficacy of treatment [67], whereas it showed the capability for parenteral drug delivery [68]. Spray-dried water-insoluble drug nanoparticles incorporated into water-soluble carrier particles improved drug absorption in rats and could be delivered via oral, pulmonary, and injection pathways [69].

The administration of pharmaceutical drugs by inhalation allows for more efficient absorption of drug molecules from the lungs, in contrast to oral, nasal, or transdermal routes [70]. Moreover, inhalation allows direct drug delivery to the target organs with minimal side effects [71]. DPIs are considered the most suitable for inhalation purposes because they are portable, economical, easy to handle, and environmentally friendly compared to nebulizers or pressurized metered dose inhalers and ensure better formulation stability than liquid dosage forms [72]. DPIs contain an active pharmaceutical ingredient of suitable aerodynamic size of 1–5 µm for inhalation [73]. Studies have suggested that the effectiveness of using spray-dried powder for the inhalation of spray-dried excipient powder enhances the clearance of mucus in the lungs [74,75]. Several researchers have suggested that for children affected by cystic fibrosis, inhaled dry powder shows remarkable improvements in lung function, regardless of age or disease severity [76,77,78,79]. Spray-dried powder showed bronchial responsiveness upon inhalation in people with asthma [80]. Spray-dried powder was found to be a suitable carrier in powder inhalers and can be produced with the desired properties by spray drying to meet the needs of the chosen drug [81]. The proportion of disintegrated nanoparticles increased with increasing excipient concentration, suggesting that a suitable excipient can convert nanoparticles into a dry inhalable powder by spray drying [82].

Spray-dried chitosan/mannitol/leucine–quercetin formulation for inhalation showed high yield (approximately 65%), encapsulation efficiency (>45%), and drug loading (12–26%) [83]. The drying temperature influenced the crystallization of the spray-dried excipients. Lower inlet temperatures (70 °C and 90 °C) lead to a slow release of moisture content and a longer crystallization period [84]. The increased inlet temperature negatively affected the aerodynamic performance of the spray-dried carrier-based formulations [85]. The particle morphology of the spray-dried inhalable carrier and protein molecules changed from spherical to irregular at high inlet temperatures, and more particle agglomerates were present in the final dry powder [86]. The yield of the excipient dry powder obtained by spray drying the aqueous excipient solution was slightly higher at lower inlet temperatures [87]. When spray-dried as a single solution and mixed with a drug, the binary carriers showed better aerosol performance than the single carriers [88]. Studies have indicated that the particle size and aerodynamic diameter of large spray-dried porous carrier particles are affected by changes in the inlet temperature, feed rate, and airflow rate. The particle size of the excipients is considered an important characteristic affecting tablet dissolution. The results showed that the specific surface area was the key factor affecting the dissolution of drug molecules produced by similar particle-size excipient grades [89]. Various amounts of mannitol were dissolved in a cyclosporine A/ethanol suspension and spray-dried at an inlet temperature of 120 °C, with outlet temperatures ranging from 46 to 59 °C, and an aspiration rate of 100%, an atomization flow rate set at 819 NL/h, and a liquid feed rate of 3.5 mL/min to produce respirable particles. The addition of mannitol to cyclosporine A improved the drug dissolution rate without affecting the particle size distribution, surface roughness, and aerosol performance [90]. Powder flowability is an important characteristic of pharmaceutical drugs that ensures a uniform tablet weight and produces tablets with consistent and reproducible features. Dry powders containing protein molecules and carrier particles prepared by spray drying exhibited a high fine particle fraction [91].

Excipients consisting of amorphous drugs show faster drug release rates than microcrystalline excipients [92]. The excipient concentration influenced the physical stability and aerosolization performance of the spray-dried protein powder. High excipient concentrations led to excipient crystallization and phase separation during storage, which negatively affected the physical stability and aerosolization performance of the spray-dried protein powder for inhalation [93].

Mathematical modeling and computational fluid dynamics offer enormous potential for optimizing spray drying process conditions, thereby making it economical and accelerating the experimental process [94,95,96].

A design of experiment (DoE) approach is often used to screen, optimize, and identify the critical and non-critical process parameters affecting the final powder properties, and several studies [97,98,99] have utilized different DoE approaches for spray drying.

At the industry level, design of experiments and other multivariate statistical approaches are commonly used to recognize critical process parameters (CPPs) and their relationship with product yield and critical quality attributes (CQAs). However, these models cannot effectively predict the effects of critical process parameters on the critical quality attributes [100]. Response surface methodology (RSM) combined with the DoE approach is being effectively utilized to optimize the process parameters required to produce pharmaceutical drugs. [101,102] However, RSM has a few limitations for nonlinear processes, such as spray drying [103]. The drawback of this approach is that it does not consider the effects of independent factors on the response variables. The model does not account for the interaction between independent variables, which might significantly affect the powder properties. A nonlinear model, such as machine learning (ML), can overcome this problem by removing the errors associated with DoE–based processes [104]. ML models illustrate the relationship between input variables and response parameters in a spray drying process by considering all the bias, systematic, and data errors for better accuracy [105]. These models can identify the optimum operating parameters, predict how variation in spray drying conditions affects the CQAs of the powder, and help in real-time monitoring of important parameters to maintain optimal drying conditions [106]. In a spray drying process, nozzle blockage or overheating can significantly affect the product quality. ML models can predict nozzle blockage to prevent interruptions during experiments and improve the process efficiency [106]. They are also used for predicting drying times, reducing energy consumption, formulation development, and developing advanced drug delivery systems [107]. However, the ML models also have a few limitations that need to be addressed. They require huge, high-quality data sets for predicting the powder properties [106]. Moreover, their effectiveness is limited by their complexity, generalizability [108], inability to evaluate new parameters, and negligence to process uncertainties [109].

The motivation behind this work was to review past and current modeling techniques for modeling the spray drying process. In the first part of the study, a general outline of the spray drying process is presented, including the transport phenomena occurring during the spray drying process and the influence of the process parameters on the properties of the final dried powder. The mathematical and computational fluid dynamics (CFD) modeling of the spray drying process, its limitations, and recommendations are highlighted in sec. 4. Then, the fundamentals of machine learning (ML) models, including the framework, hybrid ML model, introduction of relevant software, and analysis techniques, are presented in Section 5, followed by conclusions.

## 2. Transport Phenomena in Spray Drying

**Figure 2:**
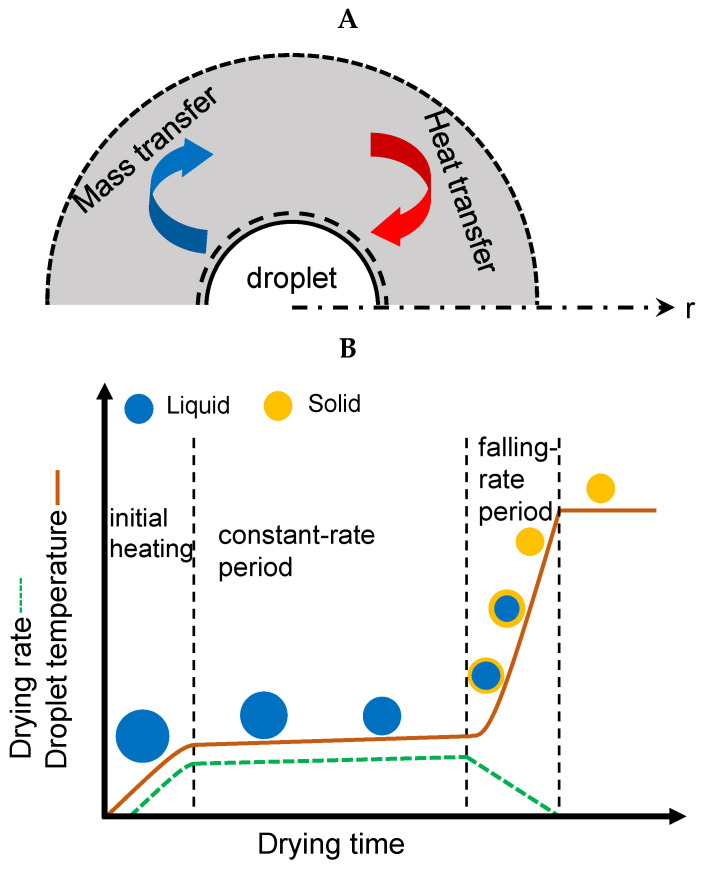
Evaporation and drying steps of a single droplet in a spray drying process: (**A**) Heat and mass transfer on the surface of the droplet; (**B**) Schematic representation of various drying stages [112].

The spray drying process begins with the atomization of the liquid feed solution into droplets, owing to a decrease in surface tension. In the atomization process, the pressurized gas enters the nozzle and breaks down the liquid into small droplets, leading to an increase in their surface area, thereby enhancing the heat and mass transfer between the hot drying gas and droplets, as depicted in Figure 2A. The size and movement of the liquid droplets through the drying chamber and their interaction with the drying gas affect the drying. Small and large droplets in any type of atomizer spray dry at different rates owing to the different periods of capillary transport, diffusion, crystallization, and heat transport. In addition, different trajectories of small and large droplets evolve owing to different drag and inertial forces, and different exposures of small and large particles to the drying gas [110]. The flow of the air-droplets inside the drying chamber can be counter-current, co-current, or mixed-flow. The co-current flow type is mostly used in the pharmaceutical industry [111].

### 2.1. Droplet Drying Process

During spray drying, the droplets encounter hot drying air; they initially heat without significant evaporation (sensible heating), as shown in Figure 2B. During this stage, the temperature increases steadily until it reaches the wet-bulb temperature [112]. Additionally, it is assumed that droplet drying occurs at a constant evaporation rate (*K*) and that the droplet diameter decreases linearly from the initial diameter (*d*_0_). This period is known as the constant drying rate period and can be described by the *d*^2^-law, $\tau_{D}=d_{0}^{2}/K$ [113]. In the spray drying process, droplet diameters are generally less than 100 µm. The value of the corresponding Biot number is normally less than 0.1 [114]. Biot number is the ratio of thermal resistance for conduction inside the droplet to the resistance for convection at the droplet surface. If the Biot number is less than 0.1, the lumped heat capacity of the droplet can be assumed, indicating that the droplet temperature is uniformly distributed. The drying temperature, humidity, and velocity of the air surrounding the droplet surface are factors that should be considered during solvent evaporation from the atomized droplets [115,116,117,118].

### 2.2. Particle Formation Process

**Figure 3:**
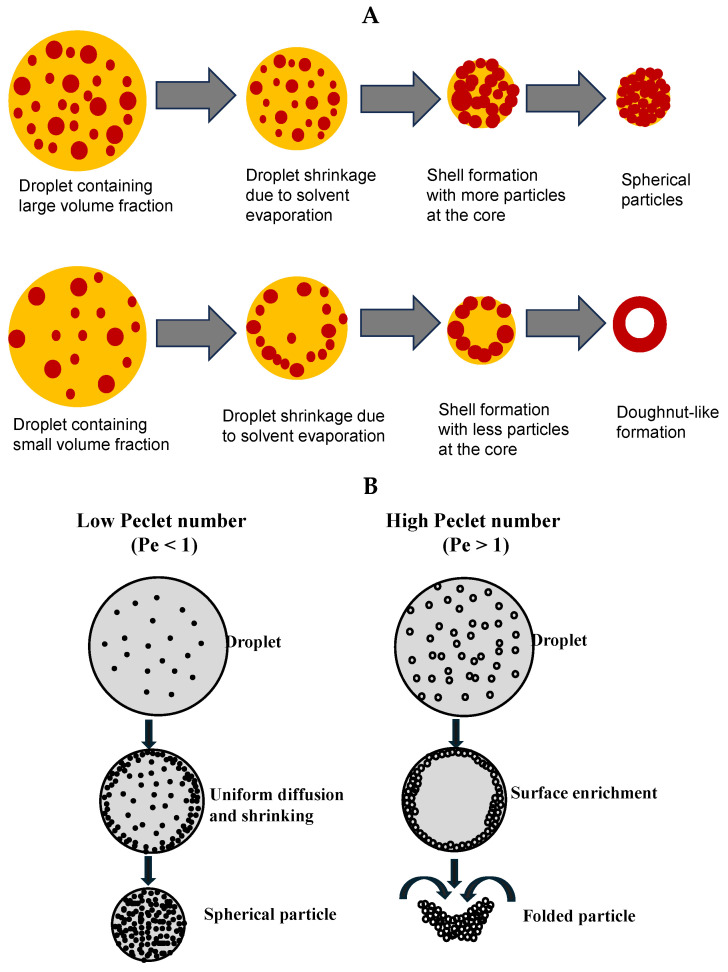
Particle formation process: (**A**) Drying of droplets containing large and small solids volume fractions; (**B**) Influence of Peclet number on final particle morphology.

The transition from a constant-rate period to a falling-rate period occurs due to the increase in solid concentration on the droplet surface. At a critical moisture concentration, the evaporation rate decreases, indicating the beginning of the falling rate period. Solute diffusion occurs inside the droplet owing to the concentration gradient between the droplet surface and the core [119]. The continuous evaporation of the solvent resulted in droplet shrinkage, and a shell began to form around the droplet surface. The vapor diffuses through the formed shell, and the particle size becomes constant. The shell significantly reduced the mass transfer of the solvent to the droplet surface. The heat transfer to the droplet during this stage increases the particle temperature. Finally, the wet particles were completely transformed into dry solid particles. A schematic of the particle formation process is shown in Figure 3A.

The morphology of the formed particles can be related to the process operating parameters using a dimensionless number known as the Peclet number (Pe), which is defined as the ratio of the evaporation rate (K) to the diffusion coefficient (D_e_). As outlined in Figure 3B, a lower Peclet number (Pe < 1) mostly leads to the formation of spherical solid particles, whereas a higher Pe value (Pe > 1) results in folded particles [120]. A high value of Peclet number, i.e., high drying temperature, results in a higher evaporation rate compared to the diffusion of primary particles in the droplet, which yields hollow particles. If the shell cannot withstand the external pressure, a folded particle is formed [121,122]. The droplet evaporation rate also influences the final particle morphology. A slow evaporation rate leads to denser particles, whereas a high evaporation rate causes skin formation on the droplet surface and produces less dense particles. Particle morphology can be explained in terms of size, shape, internal structure, and surface properties [123,124,125,126]. However, particle engineering requires a thorough knowledge of the particle formation process [127]. Therefore, it is necessary to understand and control particle formation to develop effective drug administration and particle engineering methods [127,128,129,130]. Moreover, artificial intelligence and machine learning (AI/ML) can play a significant role in bridging the influence of process parameters on the final particle morphology. ML models can predict spray-dried API particle size with errors between 7.7–18.6% [131]. Bayesian optimization can be utilized to achieve defined morphology while considering product properties such as yield, moisture, and FPF. Feature-importance/SHAP analyses indicate which input parameters (e.g., atomizing air vs. feed solids) dominantly control specific morphological outcomes, providing an advanced particle engineering hybrid method to tailor the final particle morphology of the dried powder [132,133].

## 3. Process Parameters

**Figure 4:**
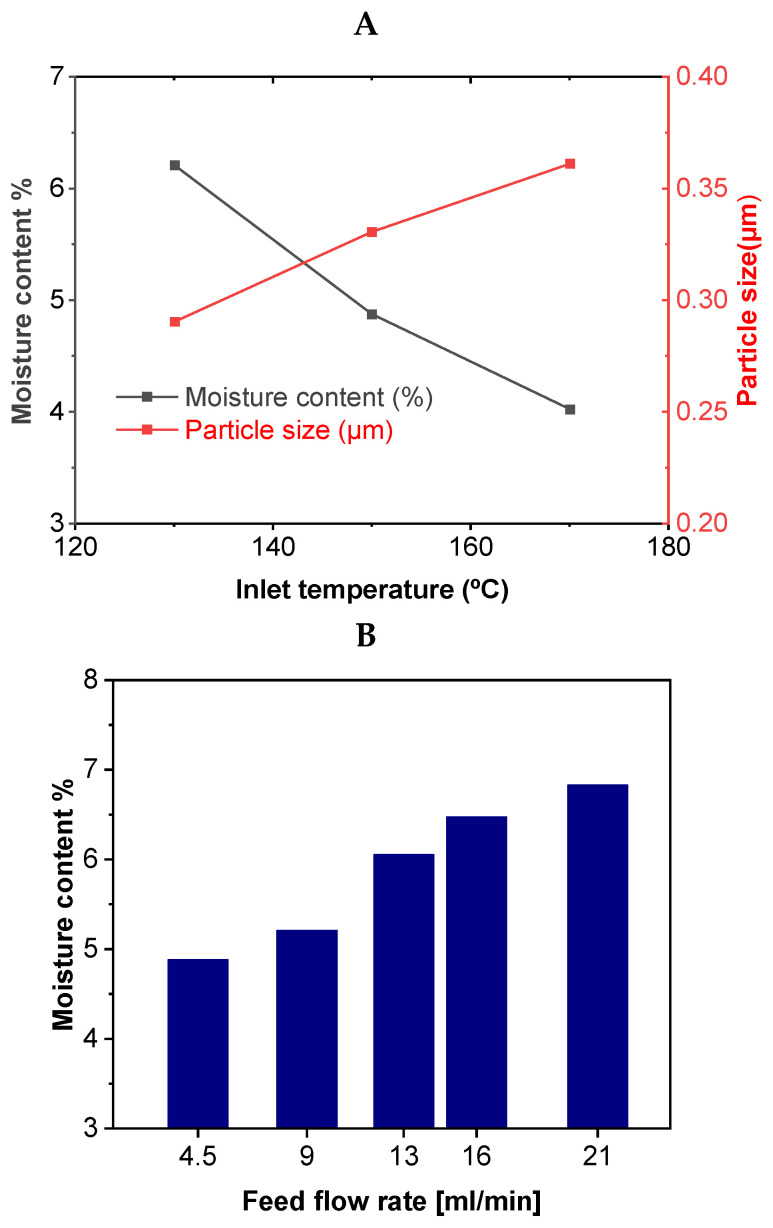
Effect of process parameters on moisture content of spray-dried powder: (**A**) Inlet temperature [144], (**B**) Feed flow rate [145].

Several process parameters, such as inlet temperature, outlet temperature, feed concentration, feed rate, drying gas rate, and spray gas flow rate, affect the final properties of the spray-dried powder. The inlet temperature significantly affects the properties and quality of the final powder. A higher inlet temperature increases the powder yield and outlet temperatures [134,135]. Moreover, a high inlet temperature influences the particle formation process owing to the high rate of solvent evaporation. A higher inlet temperature leads to the rapid formation of a solid skin on the outer surface of the droplet that captures the solvent vapors. Faster drying rates, achieved through higher inlet temperatures, result in particles with a higher Tg (glass transition temperature), making them more suitable for product stability. In contrast, lower inlet temperatures result in low Tg products with stickier particles, which reduce the final yield due to wall deposition. Inlet temperature also influences the powder properties, such as particle size, moisture content (see Figure 4A), bulk density, and solubility. The moisture gradient generated inside the droplet due to high inlet temperature can influence the particle formation process, which in turn can affect the morphology of the dried powder [136]. Variation in the inlet temperature can lead to particles of different shapes and surface roughness’s [137,138,139]. Higher inlet air temperature resulted in smoother particles and increased the breaking strength of spray-dried mannitol powder for inhalation [140]. The inlet temperature also influences the breaking strength and crystallinity of the spray-dried powders. It has been reported that a higher inlet temperature resulted in a higher degree of crystallinity for spray-dried powder [141]. The optimal inlet temperature varies depending on the sample being dried and it allows appropriate solvent evaporation and prevents product degradation [142,143].

**Figure 5:**
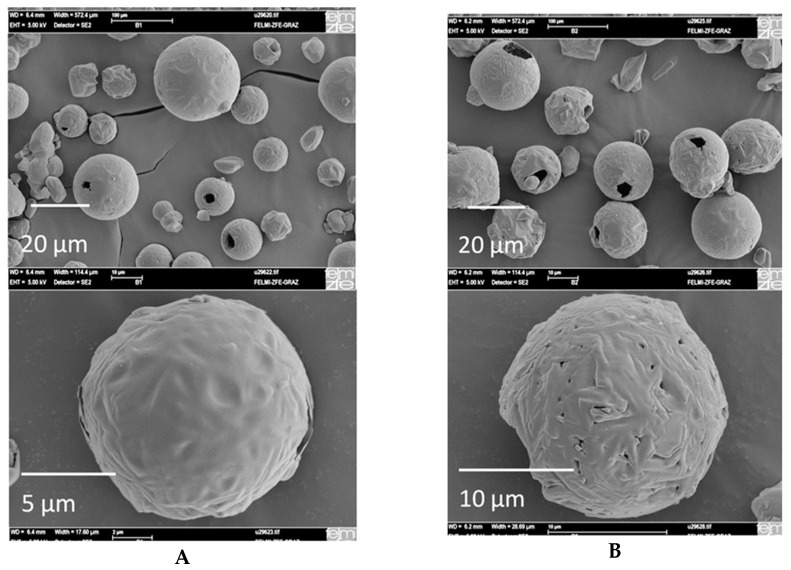
SEM images of spray-dried powder at various outlet temperatures: (**A**) outlet drying temperature at 114 °C; (**B**) outlet drying temperature at 140 °C, reproduced from [150], with permission from Elsevier, 2025.

The outlet temperature (T_out_) is a function of the feed flow rate, feed concentration, drying gas flow rate, and inlet temperature [118]. Variation in the outlet temperature did not influence the particle size, but it affected the morphology and crystallinity of the spray-dried powder, as shown in Figure 5 [146]. Reports show that when T_out_ varied between 90 °C and 157 °C, it resulted in a higher powder crystallinity [141]. Moreover, T_out_ should not be higher than the product glass transition temperature, because it increases the solid-phase crystallization rate [147]. T_out_ can also influence the residual solvent content. During droplet formation, film-producing polymers rapidly form a skin around the droplet surface, resulting in solvent entrapment, which makes solvent removal difficult [127]. Therefore, T_out_ should be maintained properly to remove the solvent content without affecting the solid dispersion stability. It has been proven that low T_out_ (60–80 °C) is needed to prevent excessive denaturation of protein [148], whereas higher T_out_ improved the storage stability of spray-dried probiotic dry powder [149].

Feed concentration can influence powder properties such as particle size, particle morphology, moisture content, and yield. It was reported that higher feed concentrations of aqueous mannitol solution resulted in particles with a rougher surface compared to lower feed concentrations. Moreover, higher feed concentrations lead to hollow particles with high porosities and low bulk densities, whereas lower feed concentrations produce smaller particles [140]. A higher feed concentration implies a lower solvent concentration in each droplet, resulting in high Pe values, shorter evaporation times, and porous, less dense particles in the final drying stage. Therefore, it increases the probability of agglomeration, leading to more porous particles with low density and rougher surfaces [151]. Also, increasing the feed concentration reduced the overall thermal efficiency, evaporative efficiency, material loss in the cyclone, powder moisture content, and bulk density, whereas increasing the particle size [152,153] and the yield of the powder.

Powder properties such as solvent evaporation rate, morphology, particle size, and density can be altered by varying the feed rate [94]. The feed flow rate is crucial because it influences the droplet size and relative humidity [154]. Higher moisture content was observed in the final dry powder at higher feed rates, resulting in a lower powder yield [151]. The effects of different feed flow rates on the moisture content of the spray-dried powders are shown in Figure 4B. Higher yields were obtained at lower feed rates. Moreover, higher feed flow rates increased the atomization of the liquid feed solution in the drying chamber and generated larger liquid droplets. As a result, moisture evaporation is difficult, and the drying efficiency is reduced [155].

Various atomization gases such as air, N_2_, Ar, and CO_2_ have been used for spray drying [156,157,158]. For liquid feed solutions containing flammable solvents, inert gases such as nitrogen are used as a drying medium to protect the products from degradation. Air is generally used as the drying gas in aqueous feed solutions.

DOE is essential in pharmaceutical spray drying for optimizing process parameters, ensuring product quality, reducing costs, supporting regulatory compliance, and enabling successful scale-up [159]. Implementing DOE in terms of factorial/response-surface designs shall overcome the limitations of the trial-and-error procedure while maintaining efficient use of limited resources and materials and revealing optimal process and formulation parameters. A summary of studies that utilized DoE in the spray drying process is presented in Table 2.

## 4. Mathematical and Computational Modeling in Spray Drying

### 4.1. Single Droplet Modeling

In the spray drying process, a single droplet was dried in two stages. In the first stage, droplet evaporation occurs, causing a decrease in the droplet diameter. In the second stage, a dry solid crust containing wet particles is produced and continuously dried until the required moisture content is achieved. Understanding the drying kinetics and mechanisms, such as evaporation, solid distribution, and skin formation, during droplet formation is essential for process control and microparticle characteristics.

The drying kinetics were modeled by solving the coupled heat and mass transfer equations. Mass coupling from the gas to the droplet occurs via evaporation, momentum transfer, and energy coupling via heat transfer. The heat and mass transfer between the droplets and the hot gas are expressed as follows:

(1) $$m_{d}C_{p}\left(\frac{dT_{d}}{dt}\right)=hA_{d}\left(T_{g}-T_{d}\right)+\left(\frac{dm_{d}}{dt}\right)h_{fg}$$

where m_d_ is the droplet mass, $C_{p}$ is the droplet heat capacity, $T_{d}$ is the droplet temperature, h is the heat transfer coefficient, $A_{d}$ is the droplet surface area, $T_{g}$ is the glass transition temperature, and $h_{fg}$ is the latent heat.

Specific equations can be used to calculate the heat transfer coefficient (h_T_) and mass transfer rate $\left(\frac{dm_{d}}{dt}\right)$. The heat transfer coefficient derived from the Ranz–Marshall equation can be written as:

(2) $$Nu=2+0.6{\left({Re}_{d}\right)}^{\frac{1}{2}}{\left(Pr\right)}^{\frac{1}{3}}$$

where Nu is the Nusselt number, k is the thermal conductivity of the gas, ${Re}_{d}$ the Reynolds number, and Pr is the Prandtl number defined as:

(3) $$Pr=\frac{C_{p}\mu }{k_{ta}}$$

Equation (4) gives the mass transfer rate for evaporation between the droplet and gas.

(4) $$\frac{{dm}_{d}}{dt}=-k_{c}A_{d}\left(Y_{S}^{*}-Y_{g}\right)$$

where $\frac{{dm}_{d}}{dt}$ is the mass transfer rate, $Y_{S}^{*}$ is the saturation humidity, $Y_{g}$ is the gas humidity, and $k_{c}$ is the mass transfer coefficient obtained from the Sherwood number (Sh):

(5) $$Sh=\frac{k_{c}d_{d}}{D_{g}}=2+0.6{\left({Re}_{d}\right)}^{\frac{1}{2}}{\left(Pr\right)}^{\frac{1}{3}}$$

where Sh is the Sherwood number, $D_{g}$ is the diffusion coefficient of water vapor in the gas phase, and Sc is the Schmidt number (Sc).

(6) $$Sc=\frac{\mu }{\rho_{g}D_{g}}$$

where μ is the molecular viscosity of the fluid and $\rho_{g}$ is the density of the fluid.

**Figure 6:**
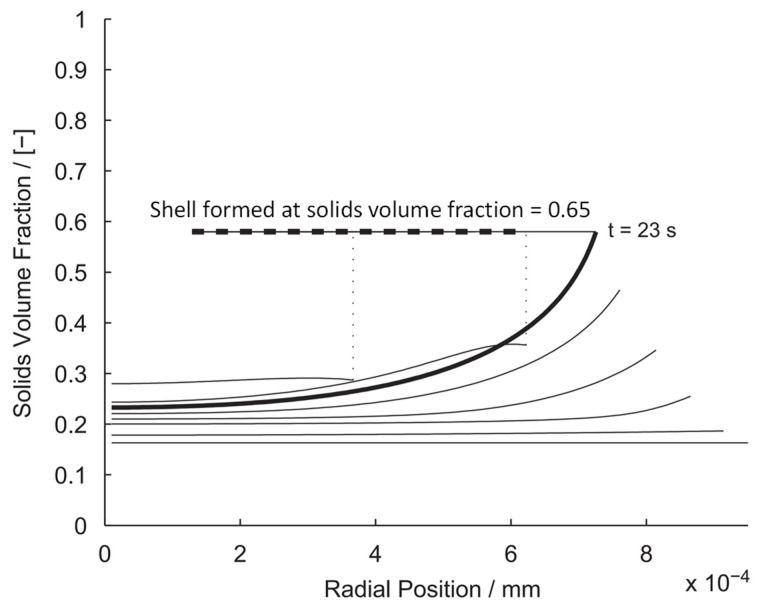
Shell formation predicted at solids volume fraction = 0.65, time = 60 s. by the model, reproduced from [179] with permission from Elsevier, 2025.

During drying, a droplet may contain either insoluble or dissolved solids. There are some important factors, such as initial droplet heating in the first drying stage, temperature profile within the droplet, heat absorption by the crust region, crust resistance to diffusion mass transfer, and temperature dependence of the physical properties, that need to be considered for modeling the drying process of a droplet. A model was developed to simulate the drying of droplets containing suspended solids for the prediction of particle morphology, along with the droplet drying rate [179]. A population balance approach was used to model the discrete solid phase by assuming all particles are spherical. This approach predicts the critical solids volume fraction at which the shell forms, as illustrated in Figure 6. From the nature of the shell formed, particle morphology can be determined, as a shell formed from particles with a similar diameter will have a higher porosity than one consisting of particles of different sizes. In another study [180], an experimental technique using an acoustic levitator was carried out to examine the single droplet drying kinetics of spray-dried PLGA/ethyl acetate microparticles. The results presented the influence of process variables such as inlet temperature (0–40 °C), polymer concentration (5–400 mg/mL), and droplet size (0.5–1.37 μL) on the drying time and drying kinetics, as well as the particle morphology.

**Figure 7:**
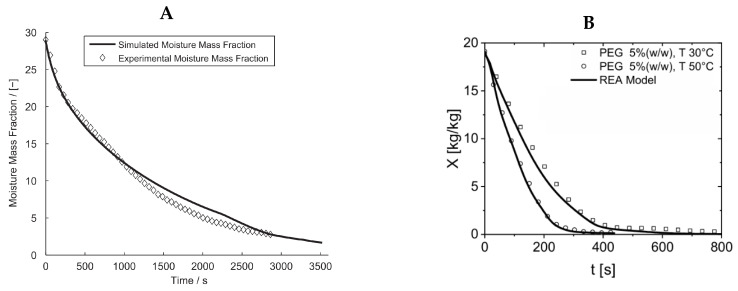
Drying of a single droplet: (**A**) temporal evolution of moisture mass fraction (simulation versus experimental results) [179] with permission from Elsevier, 2025; (**B**) droplet drying curves of Polyethylene glycol 6000 in terms of dry mass content (X) versus time, theoretical prediction of the REA model versus experiments [188].

Previous studies mainly modeled the drying kinetics based on the average moisture content and neglected these factors [181,182]; however, a more advanced and realistic theoretical model that considered all these factors was developed, and it showed good agreement with experimental measurements, as shown in Figure 7A [183]. In a recent study, the single droplet drying kinetics of maltodextrin were investigated using experimental and numerical techniques. The experiments analyzed the variations in critical moisture content, and the model was employed for use in simulations of spray drying. However, the model accuracy was reported to be low [184]. Due to the low model accuracy of the CFD model, the optimized spray drying tower is expected to have uneven temperature distribution and particle trajectory, resulting in high wall deposition and low product quality. Therefore, a New CFD model (N-CFD) was developed to simulate the temperature distribution and particle trajectory in a spray drying chamber based on experimental data. Simulation results proposed a new type of air inlet mechanism that can even out the temperature distribution and particle trajectory in the drying chamber [185]. CFD simulations were also carried out in the food industry to analyze the effect of milk performance parameters on the spray dryer conditions. Results showed that increasing both the milk mass flow rate and the diameter of the particles resulted in an increase in particle velocity and a decrease in both the inlet temperature and outlet temperature of the particles [186].

Recently, a successful lumped drying model, the Reaction Engineering Approach (REA), was developed to dry individual droplets containing dissolved solids [187,188]. The REA model postulates that moisture must overcome an energy barrier to migrate from the interior to the exterior of the droplet surface. According to this approach, the partial vapor pressure over the surface of a droplet with dissolved solids is not only a function of temperature but also of the droplet’s moisture content [189]. The REA model was used to predict drying kinetics in terms of droplet size, shape, and morphological changes during the drying process. The developed model was then compared with experimental measurements. As depicted in Figure 7B, the droplet drying curves of both the REA model and experimental data are in good agreement.

### 4.2. CFD in Spray Drying

CFD simulations are commonly used in spray drying to model and optimize the design parameters, thereby enhancing the product quality [190,191,192,193,194]. The complex nature of the spray drying process includes multiple physical phenomena, such as liquid atomization, droplet interactions, agglomeration and coalescence of the particles, and mass and heat transfer on the scale of single droplets. Using CFD, we can predict the gas flow patterns, temperature and velocity distributions, particle trajectories, and humidity within the drying chamber [190,195,196,197].

**Figure 8:**
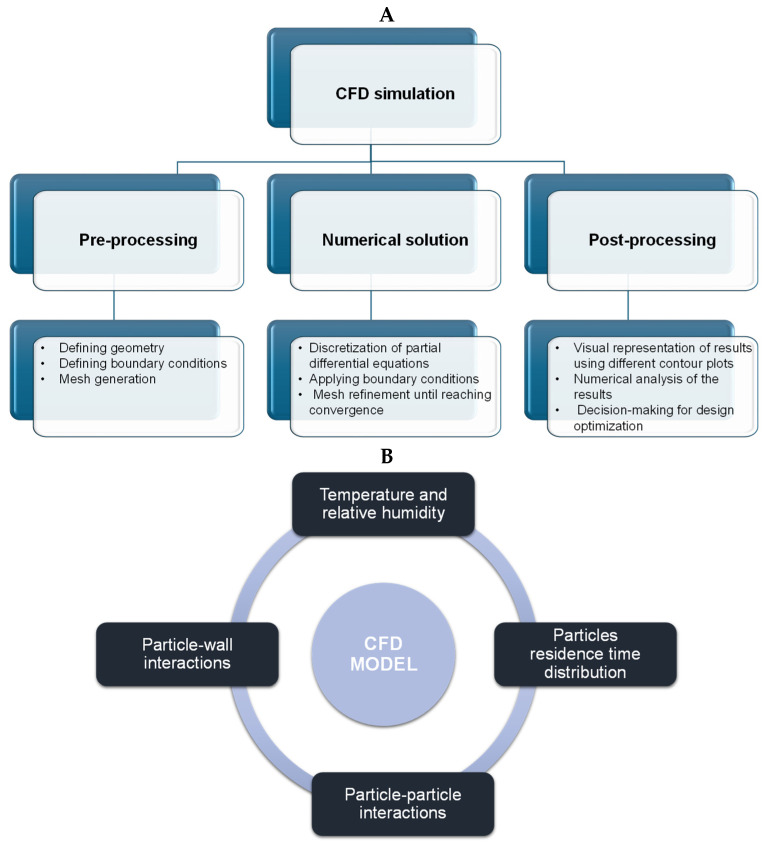
Computational fluid dynamics (CFD) simulation workflow: (**A**) Stages involved in CFD Analysis; (**B**) Parameters obtained in the spray drying process using CFD simulation, adapted from [198], with permission from Elsevier, 2025.

**Figure 9:**
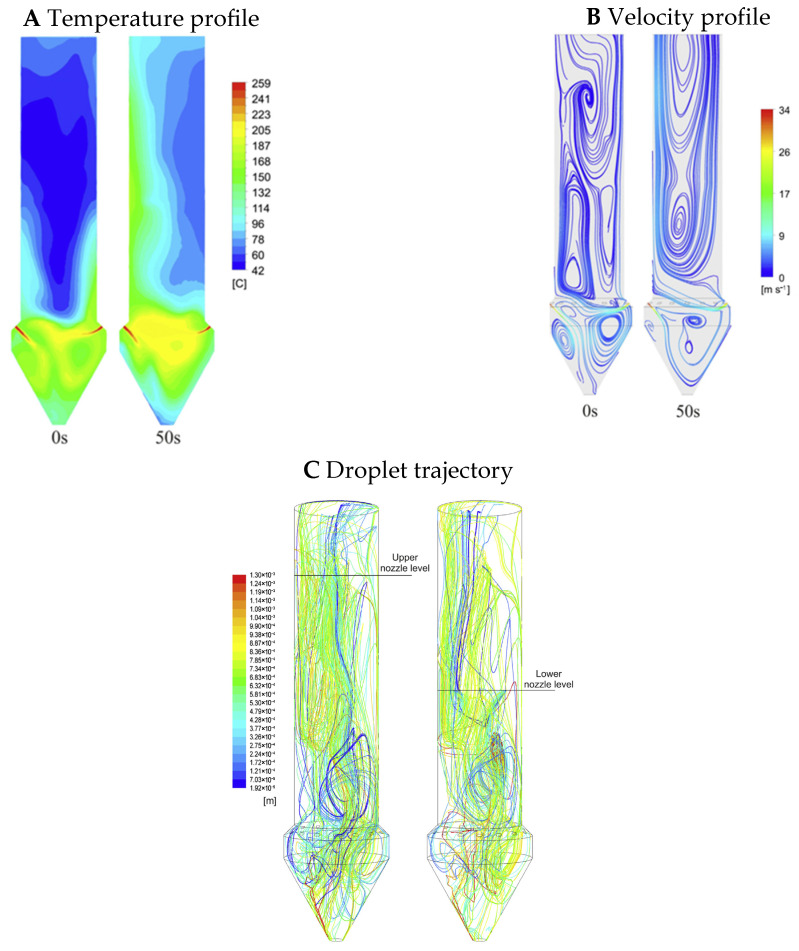
CFD simulation of droplet drying in spray dryer: (**A**) 2D temperature profiles; (**B**) velocity profiles for continuous phase transport in a spray dryer; (**C**) droplet trajectory for average evaporation rate. Reproduced or adapted from [209], with permission from Elsevier, 2025.

CFD analysis involves three stages: preprocessing, numerical simulation, and postprocessing. In the first stage, the geometry and boundary conditions are defined, and a mesh is generated for the drying chamber [198]. During the processing stage, partial differential equations are discretized using finite element, finite difference, or finite volume methods. Boundary conditions are applied, and the resulting algebraic equations for each mesh are numerically solved using Gaussian elimination or the Gauss–Seidel method are numerically solved. The residual errors between consecutive iterations are minimized until convergence is achieved. The final stage involves the visualization of the simulation results using different contour plots that allow us to make decisions for design optimization. All the stages involved in the CFD analysis are shown in Figure 8A. During the spray process, phenomena such as particle–particle collisions and particle–wall interactions can be observed inside the drying chamber, as depicted in Figure 9B. Accurate prediction of particle–particle collisions is important, as it might affect the size and morphology of the final dried powder. Conversely, particle–wall collisions result in a low powder yield owing to the deposition of particles on the walls of the drying chamber. The particle residence time distribution is crucial because of the heat-sensitive nature of some products. Table 3 presents an overview of the use of CFD modeling in a wide range of applications.

The spray drying process involves a gas phase (drying air), liquid phase (droplets), and solid phase (particles). Because the gas phase occupies more volume than the liquid and solid phases during spray drying, it can be assumed to be a continuous phase, whereas the solid and liquid phases can be considered discrete phases. The velocity and temperature profiles for the continuous phase transport can be predicted using CFD simulation, as illustrated in Figure 9A,B. For the continuous phase, the conservation of mass, momentum, energy, heat, and mass transfer, temperature, turbulence kinetic energy, and dissipation rate of kinetic energy are expressed as [208]:

**Continuity equation:**

(7) $$\frac{\partial \rho }{\partial t}+\frac{\partial }{\partial x_{i}}\left(\rho u_{i}\right)=0$$

where ρ is the density, x is the cartesian coordinate, u is the velocity, and i is the cartesian coordinate index.

**Momentum equation:**

(8) $$\frac{\partial }{\partial t}\left(\rho u_{i}\right)+\frac{\partial }{\partial x_{j}}\left(\rho u_{i}u_{j}\right)=\frac{\partial }{\partial x_{j}}\left[-p\delta_{ij}+\mu \left(\frac{\partial u_{i}}{\partial x_{j}}+\frac{\partial u_{j}}{\partial x_{i}}\right)\right]+\rho g_{i}$$

where δ is the Kronecker delta, µ is dynamic viscosity, and g is the acceleration due to gravity.

**Energy conservation:**

(9) $$\frac{\partial }{\partial t}\left(\rho C_{p}T\right)+\frac{\partial }{\partial x_{j}}\left(\rho u_{j}C_{p}T\right)-\frac{\partial }{\partial x_{j}}\left(K\frac{\partial T}{\partial x_{j}}\right)=s_{T}$$

where C_P_ is the specific heat capacity, T is the temperature, K is the thermal conductivity, and s_T_ is the thermal source.

The differential equation of heat transfer is used to determine the energy transfer and is given by:

(10) $$\frac{\partial }{\partial t}\left(\rho C_{p}u_{i}\right)=\frac{\partial }{\partial x_{j}}\left(\lambda \frac{\partial T}{\partial x_{j}}\right)+s_{T}$$

Fick’s law of mass diffusion can be used to describe mass transfer and is expressed as follows:

(11) $$\frac{\partial X_{W}}{\partial t}=\frac{\partial }{\partial x_{j}}\left(D_{eff}\frac{\partial X_{W}}{\partial x_{j}}\right)$$

where D_eff_ is the effective diffusion coefficient.

**Turbulence kinetic energy:**

(12) $$\frac{\partial }{\partial t}\left(\rho k\right)+\frac{\partial }{\partial x_{j}}\left(\rho u_{j}k\right)=\frac{\partial }{\partial x_{j}}\left(\frac{\mu_{e}}{\sigma_{k}}\frac{\partial k}{\partial x_{j}}\right)+G_{k}+G_{b}-\rho \varepsilon$$

**Dissipation rate of turbulence kinetic energy:**

(13) $$\frac{\partial }{\partial t}\left(\rho \varepsilon \right)+\frac{\partial }{\partial x_{j}}\left(\rho u_{j}\varepsilon \right)=\frac{\partial }{\partial x_{j}}\left(\frac{\mu_{e}}{\sigma_{\varepsilon }}\frac{\partial \varepsilon }{\partial x_{j}}\right)+\frac{\varepsilon }{k}\left(C_{1}G_{k}-C_{2}\rho \varepsilon \right)$$

For the discrete phase, particle/droplet movement is expressed by Newton’s Second Law:

(14) $$\frac{dU_{p}}{dt}=g+\frac{\sum F}{m_{p}}$$

Here, ∑F is the sum of the forces (drag force (F_D_), buoyant force (F_B_), contact force (F_c_), and added mass force (F_A_) acting on a given spray particle by the gas phase and other particles and walls of the spray drying chamber in Equation (14).

In the spray drying process, CFD mostly uses the Eulerian–Lagrangian method because it calculates the residence time of individual droplets within a wide range of droplet sizes [210]. In the CFD simulation, an Eulerian–Lagrangian model was used to calculate the droplet trajectories for different evaporation rates, as shown in Figure 9C by solving the force balance in Equation (15):

(15) $$\frac{{du}_{d}}{dt}=\left(\frac{18\mu }{\rho_{d}d_{d}^{2}}\right)\left(\frac{C_{DR_{e}}}{24}\right)\left(v-u_{d}\right)+g\left[\frac{\rho_{d}-\rho_{g}}{\rho_{d}}\right]$$

where $u_{d}$ is the droplet velocity, $\rho_{d}$ is the droplet density, $d_{d}$ The droplet diameter, $C_{D}$ is the drag coefficient, R_e_ is the Reynolds number, v is the fluid phase velocity, g is the gravitational force, and $\rho_{g}$ is the density of the fluid.

The droplet temperature is crucial for heat-sensitive products because it affects the thermal stability of heat-sensitive components. CFD simulation was used in the spray drying process to study the effect of droplet temperature in both short-form and tall-form spray dryers [210]. The droplet residence time significantly affects the critical quality attributes of the final powder, such as the solubility and bulk density. The droplet residence time in a superheated spray dryer was experimentally calculated and validated using CFD predictions. It has been reported that droplet residence time is significantly affected by droplet size and operating parameters [211]. Another study conducted in a short- and tall-form spray dryer suggested that most droplets had a low residence time during the spray drying process [210]. The droplet impact on the drying chamber is also significant for the design and operation of spray dryers and for improving the powder quality. The droplet impact positions were different for the short- and tall-form spray dryers. In the short-form spray dryer, a large proportion of the droplets struck the conical part of the drying chamber, whereas in the tall-form spray dryer, the droplets struck the cylindrical part [190].

Some of the CFD models for the spray drying process apply 3D analysis, an unsteady-state model, and a population balance approach to simulate the droplet drying mechanism inside the spray drying chamber to predict the asymmetry of the flow patterns inside the drying chamber [212]. Transient model calculations conducted for droplet–droplet interactions showed that droplet collisions affected temperature and humidity patterns, whereas their impact on velocity was less notable [213]. The population balance model explains droplet growth, coalescence, and breakup during spray drying [210]. It is frequently used to analyze dispersed systems such as colloids, polymers, and aerosols, and to track particle properties and their changes due to aggregation, fragmentation, nucleation, and growth in spray drying processes and various other fields [214].

The velocity profile, heat transfer, and air flow inside the spray drying chamber are extensively modeled using ANSYS Fluent 2025 R2 (commercial) and OpenFOAM v2506 (open source) platforms. These platforms provide high accuracy in turbulent modeling and have the capability to address domains with high spatial resolution, making them appropriate for detailed simulations in both academic and industrial environments. ANSYS Fluent is known for its user-friendly interface and robust physical models, making it accessible for users with little programming knowledge [215,216,217]. Nevertheless, it requires considerable resources and knowledge to function effectively. OpenFOAM is associated with a harder learning curve and generally requires more programming knowledge and skills to operate properly [216,217]. The key features of both platforms are summarized in Table 4.

### 4.3. Limitations and Recommendations in CFD Modeling

CFD modeling has several advantages, such as the prediction of temperature and velocity distribution, particle size, relative humidity, and several other factors of the spray drying process that influence the final properties of the dry powder [210]. However, there are also a few limitations owing to the coexistence of multiple phases (solids and fluids) in the spray drying process. In CFD spray drying simulations, model assumptions affect prediction accuracy. The choice of gas-phase turbulence model, steady-state or transient, and the selection of 2D axisymmetric vs. full 3D domains shall influence residence times, mixing, and recirculation patterns. Mesh discretization (including mesh quality and mesh size) and thermophysical simplifications (e.g., lumped thermal properties, neglected heat losses) further impact temperature and vapor distribution profiles. Reported maximum errors in discrete-phase predictions can approach 20% in such models [227]. Additional contributors include the treatment of particle–turbulence dispersion, atomizing air, initial atomization parameters (droplet size/velocity distributions), and wall heat-loss modeling [227,228]. It is difficult to predict the mass transfer that occurs within a particle during the spray–drying process using CFD modeling without the help of surrogate models that consider mass transfer, particle collisions, stickiness, and agglomeration. Moreover, experimental validation of CFD results is complicated and, to a certain extent, impossible owing to the limitations of experimental measurements [198,229], including a lack of adequate data on the physicochemical properties of materials, difficulty in determining the drying kinetics during the spray drying process, shrinkage during the drying process resulting in complex mesh generation, longer computational times owing to different time scales of fluid flow, heat, mass, and scalar transport, and different particle trajectories may arise owing to the circulation of drying air inside the drying chamber.

The drying kinetics and droplet shrinkage during the spray drying process are difficult to model using CFD modeling. A reduced-order model based on a population-based approach is an innovative method to accurately capture droplet shrinkage and drying kinetics. The main advantage of this approach is that it reduces computational costs, allowing it to simulate the entire production process. Furthermore, it uses the initial droplet size distribution to characterize the granules, thereby reducing the numerical errors due to discretization. The application of the population balance model in the spray-drying process is still limited. However, owing to its computational efficiency, it is recommended that the model accuracy be improved by including the droplet trajectory information from previously solved CFD simulations [212]. Particle size distribution is crucial in the spray-drying process. Droplet coalescence and particle agglomeration influence particle size distribution. Incorporating empirical expressions into an agglomeration/coalescence CFD model is an efficient way to accurately predict the particle size. To achieve this, existing binary collision models were modified and used in CFD together with a second adhesion model that involved more realistic droplet–particle, particle–droplet, and particle–particle collisions for efficient prediction of droplet–droplet collisions. Research studies [230,231] suggest that the proposed models can predict particle growth and final particle size with good accuracy and locally investigate the spray–drying process in different parts of the drying chamber. The main advantage of this CFD approach is that it considers heat loss and drying kinetics, along with particle agglomeration and coalescence. Studies related to droplet–droplet collisions have assumed only droplet coalescence for the prediction of particle agglomeration. Nevertheless, the prediction accuracy of the current model can be enhanced by further improving the sub-models for adhesion, formation of different droplet sizes, and particle breakage [230].

Accurate prediction of the residence time distribution (RTD) of droplets inside the spray drying chamber is crucial, as it influences the final powder properties. However, there is a lack of direct validation of experimental measurements with the CFD model, particularly in a counter-current spray dryer. Therefore, it is recommended to conduct specific studies that directly compare the measured and predicted RTD of droplets inside the drying chamber for enhancing the CQAs of the spray-dried powder [232]. Many CFD models assume that droplet shrinkage occurs because of water loss, thus neglecting the expansion/contraction of droplets due to trapped gases. These simplified assumptions can affect the prediction accuracy related to droplet drying kinetics and particle formation [233]. It is suggested to use more advanced drying kinetics models that consider the contraction and expansion of droplets for a more accurate representation of the particle formation process [228].

DEM combined with CFD was used to evaluate the role of surface tension on granule formation in a spray drying process. A CFD-DEM model containing a capillary force law based on Young–Laplace and Young equations was derived for particles in contact with liquid surfaces. Simulation results suggested that a non-linear correlation exists with subsequent granule density and morphology [234]. A coupled unresolved CFD-DEM approach was recently used for numerical simulation of the solidification of the single droplet suspension in a spray drying process. In this model, the Discrete Element Method (DEM) solves the equations of particle trajectory, while Computational Fluid Dynamics (CFD) computes the interstitial fluid flow [235]. The average drying conditions for a single droplet were obtained from a simulation of a large-scale spray dryer for different droplet sizes. The results showed that appropriate process parameters in a spray dryer can be determined using CFD-DEM simulation for the formation of particles with specific morphology [236].

A multi-scale multiphase approach was used to model the transport phenomena in a spray drying process based on experimental and theoretical data. The model utilized an Eulerian–Lagrangian approach and was solved numerically using the CFD technique. Successful validation and prediction of drying behavior of single droplets of suspensions, and two- and three-dimensional steady-state calculations of spray-drying processes were reported [237]. Another multi-scale approach that combines molecular-level packing interpretation with a continuum diffusion model was developed to predict the effect of initial droplet size on spray-dried protein-lactose powder. Model predictions pointed out that both the mean droplet size and the standard deviation of the log-normal size distribution had a huge influence on the surface composition of the particles [238].

Longer computational times owing to the different time spans of fluid flow, heat, mass, and scalar transport make the simulation process expensive. Previous studies [239] focused on reducing computational time by controlling the grid resolution by region. This approach focused only on the unsteady nature of the flow in different regions, and the grid resolution requirements changed dynamically within the domain. Therefore, using an advanced mesh refinement (AMR) strategy is an effective way to perform cost-effective transient simulations of the spray-drying process [240]. The use of AMR significantly reduced the computational cost compared to the base case (fixed grid with two refinement levels) and helped capture large eddies in critical regions. This approach provides a proper balance between solution accuracy and computational cost. Owing to the low computational cost involved, it is recommended to carry out parametric studies of the spray drying process, where computational expenses are a major limiting factor.

Several challenges in CFD simulations of spray drying can be addressed by implementing machine learning-aided models, hybrid models, and computation-efficient methods aided by machine learning. In the next section, the use of machine-learning-aided models to address the limitations of CFD models in the spray drying process is discussed in detail.

## 5. Machine Learning-Based Predictive Models

Machine learning (ML) techniques have recently emerged as integral tools for data analysis, predictive modeling, and decision-making in a wide range of engineering applications [241,242,243,244,245,246,247]. ML models can process large amounts of diverse data without vast computational resources [248]. To construct models that can generate accurate predictions, machine learning algorithms are trained on labeled datasets [249]. Important factors affecting model performance are the quality and reliability of the training data.

The core of the ML method comprises statistical and computational algorithms that analyze data, learn from it, and make predictions or decisions. The accuracy of the ML model is assessed quantitatively using performance metrics that determine the prediction level of the trained model [250].

### 5.1. Machine Learning Framework

**Figure 10:**
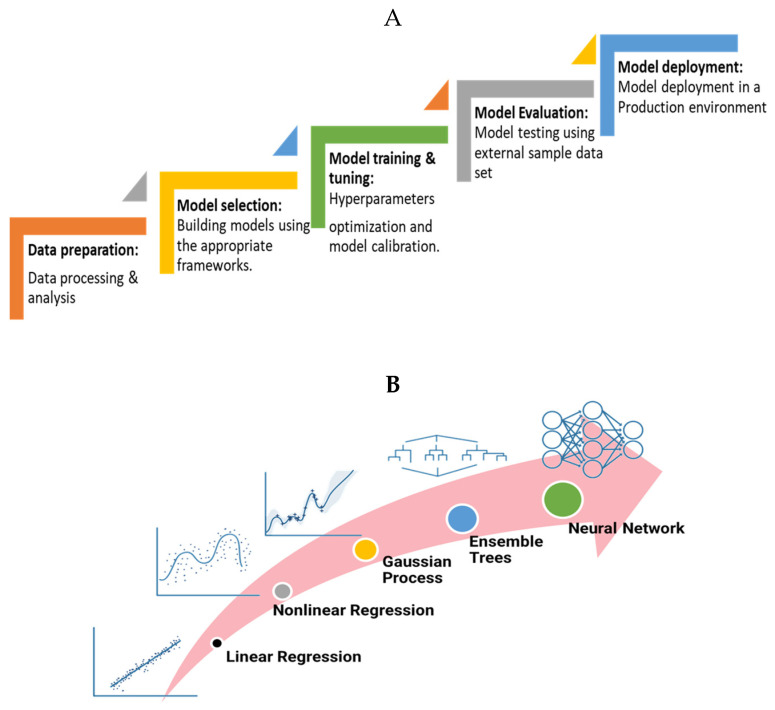
Machine learning workflow: (**A**) Steps involved in developing AI/ML-based models; (**B**) Different levels of complexity of ML models, ranging from simple linear regression models to more complex neural networks.

The development of ML-based models consists of different stages, beginning with data preparation for model deployment, as depicted in Figure 10A. In the first stage, a dataset representing the studied problem–experimental or simulation trials–is prepared [251]. The collected dataset is then validated to evaluate the data quality for building an optimal ML model [252]. Second, different ML models are compared in terms of their prediction accuracy. In the third stage, hyperparameter tuning algorithms are applied to the trained model to obtain the optimal hyperparameters that generate the best prediction accuracy [253].

Subsequently, the model is evaluated by creating a confusion matrix to study the performance parameters such coefficient of determination (R^2^) and root mean squared error (RMSE). Finally, the best ML model was integrated into engineering applications. Regression algorithms were evaluated using metrics such as the coefficient of determination (R^2^), mean squared error (MSE), and root mean squared error (RMSE). A smaller RMSE value indicates better predictive accuracy:

**Coefficient of Determination (R^2^):**

(16) $$R^{2}=1-\frac{\sum_{i=1}^{n}{\left(y_{i}-\hat{y_{i}}\right)}^{2}}{\sum_{i=1}^{n}{\left(y_{i}-\bar{y}\right)}^{2}}$$

**MSE:**

(17) $$\mathrm{MSE}=\frac{1}{n}\sum_{i=1}^{n}{\left(y_{i}-\hat{y_{i}}\right)}^{2}$$

**RMSE:**

(18) $$\mathrm{RMSE}=\sqrt{\frac{1}{n}\sum_{i=1}^{n}{\left(y_{i}-\hat{y_{i}}\right)}^{2}}$$

where $y_{i}$ is the actual value, $\hat{y_{i}}$ the predicted value, $\bar{y}$ the mean of the actual values, and n the number of observations.

Various machine-learning algorithms are depicted in Figure 10B, ranging from basic linear regression to complex neural networks. The linear regression model is the simplest data-based ML model and is also referred to as the least-squares-based method [254], which is a direct method that predicts the response variable Y based on a single predictor variable X. It was assumed that a linear relationship exists between X and Y. If more than one predictor variable is present, the model is known as a multiple regression model.

Nonlinear regression models are frequently used for nonlinear relationships in complex systems [255]. Linear Support Vector Machines (SVMs) are supervised by ML algorithms that classify data by finding an optimal line or hyperplane that maximizes the distance between classes in an N-dimensional space [256,257]. They have been widely used in ML because they can handle both linear and nonlinear regressions and classifications [258,259]. SVMs allow for the generalization of new data and accurate classification and regression predictions.

Gaussian process models are generally used to carry out Bayesian nonlinear regression and classification and are integral to many machine-learning problems [260]. These models can model complex data relationships and identify high-level data properties, such as inputs, that are critical for identifying the response. Ensemble trees are another ML model that improves classification accuracy by combining multiple decision trees. It also addresses the overfitting and variance issues [261]. Variance refers to the sensitivity of a model to small changes in the training data. A high variance leads to overfitting of the target, whereas a low variance results in underfitting. Ensemble tree models include random forest, rotational forest, and extremely randomized trees [262]. Gradient Boosting Regression is another ensemble ML technique used for classification and regression. It builds a series of decision trees, and each new tree attempts to correct the errors made by previous ones. The final prediction was made by combining the outputs from all the trees [263].

**Figure 11:**
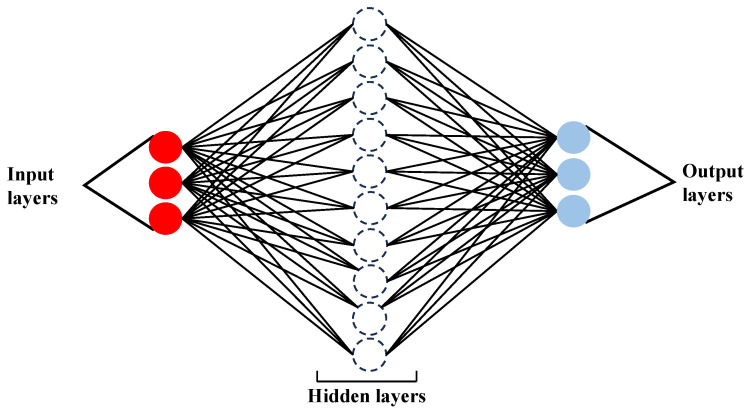
Schematic representation of an Artificial neural network (ANN).

Neural networks are ML-based empirical modeling techniques that can approximate continuous nonlinear relations [264]. These are the most complex ML models and contain artificial neurons that process and transmit signals [265]. They were trained using backpropagation and gradient descent methods, which contained strategies to eliminate overfitting. The different types of neural networks include feedforward, convolutional, and recurrent neural networks. Among neural networks, artificial neural networks (ANNs) are the most general type of neural network that can be used for different tasks such as classification, regression, and pattern recognition. ANNs have been studied in recent years and have shown better approximations than the RSM [266]. An artificial neural network is a computational method that develops data, such as data from the human brain [267]. It consists of an input layer that receives the initial data, an intermediate layer consisting of a complex structure of neurons (hidden layers), where complex mathematical calculations occur [268], and an output layer that produces results for the given input data. A schematic of the ANN is shown in Figure 11.

ML models have shown enormous potential in recognizing new CPPs that can effectively predict CQAs [100]. An overview of the use of ML models for predicting the CQAs of spray-dried powders is presented in Table 5.

The ML approach is used in various engineering applications and can be used to produce final biopharmaceutical products [269] and pharmaceutical spray drying [270,271,272,273]. In pharmaceutical drug delivery, ML plays a crucial role in improving drug solubility and bioavailability while minimizing experimental measurements. It utilizes huge experimental datasets and data–driven supervised algorithms for drug formulation optimization. In case of limited experimental data for model training, statistical learning models were employed to predict the properties and phenomena occurring between drug particles and carriers [274]. Multiple linear regression models were used to predict the long-term physical stability of poorly water-soluble drugs [275]. Recently, ML models such as ANNs have been prominently utilized in drug development for predicting and optimizing the drug composition, stability, and dissolution rates [276,277]. The particle size of the spray-dried powders is an important property affecting the bioavailability of the final drug product. An ensemble machine-learning model showed how the variation in process variables for a given pharmaceutical drug and formulation impacted the spray-dried particle size. In a recent study, ML models have been used for both experiments and image classification to examine the aerosol performance of dry powders during inhalation [269]. The powder yield is important for scale-up, and the median particle size distribution is crucial for processing and product design. Ensemble artificial neural networks can predict the powder yield and median particle size distribution of polymer-based amorphous solid dispersions [278]. Moreover, the ML approach has been successfully employed to optimize 3D-printed tablets [279], predict the fine-particle fraction and emitted dose of dry powder for inhalation [280], determine tablet defects [281], and determine the physical stability and dissolution rate of solid dispersions. The production of pharmaceutical drugs as amorphous formulations is considered a feasible method for improving the solubility of recent drug formulations [282]. However, amorphous solids tend to crystallize over time, which is difficult to predict. ML algorithms can be used to predict the physical stability of amorphous drugs [283]. Optimizing formulation parameters is crucial for ensuring the quality of liposomal nanoparticles [284]. Nevertheless, optimizing these parameters through experimentation is expensive and time-consuming [285]. ML models, through accurate predictions, reduce the cost and time associated with the spray drying process.

**Table 5:** ML models used to predict critical quality attributes of spray-dried powder.

Machine Learning Models	Input Parameters Studied	Critical Quality Attributes Predicted	Refs.
**Ensemble machine learning (EL)**	Sonication time, extrusion temperature, and feed composition	Particle size, and polydispersity index (PDI) of liposomal particles, in vitro dissolution profile	[284,286]
**Artificial neural network**	Different types of drugs and excipients, carrier concentration, particle size, and morphology	Drug–excipient interactions, powder yield, emitted dose, fine particle fraction	[280,286,287]
**Support Vector Machine (SVM)**	Effects of the type of core and shell materials and their concentrations, effect of particle size	In vitro dissolution profile of sustained-release tablets, tablet tensile strength, and tablet brittleness index	[286]

ML model accuracy can be improved by combining predictions from various learning models and provides a better assessment of optimum formulation parameters. A summary of the prediction accuracy of different ML models is given in Table 6.

Recently, ML has been suggested to assist in the Quality by Design process [291]. Further, experimental designs play a vital role in producing high-quality training datasets for ML models in the spray drying process. DoE offers systematic data collection and information related to the interaction between variables, whereas ML provides improved prediction capacity for process optimization and design space development. It has been proposed that predictive models developed based on DoE datasets using different ML algorithms, such as the Backpropagation Neural Network (BPNN), genetic algorithm-based BPNN, mind evolutionary algorithm-based BPNN, and Extreme gradient boosting. Machine have helped to understand the impact of critical process parameters that affect the final properties of the powder [292]. A combination of DoE and artificial neural networks as shown in Table 7 gave superior prediction performance compared to the traditional response surface methodology [293]. In another study, DoE was performed with a surrogate material such as α-lactose monohydrate instead of the actual protein. This was done to minimize the cost associated with the proteins. The prediction results of DoE and ML models were compared with three protein-based validation runs. Within the design space investigated, the powder yield, residual moisture content, and protein secondary structure were found to be satisfactory [294]. A thermodynamic model based on experimental points from the QbD approach was developed and compared with a CFD model to estimate the outlet temperature. The experimental points from the QbD approach were used to create the dataset for the thermodynamic model. The thermodynamic model utilized an ML approach to overcome the drawbacks of the lab-scale spray dryer. As a result, it showed a more accurate prediction of outlet temperature compared to the CFD model [295].

Data analysis, building, and deployment of ML models are carried out using ML platforms like TensorFlow 2.20.0 (open source) and MATLAB R2025a (commercial). TensorFlow is known for its robust and effective deep neural network (DNN) implementations. It is utilized to train advanced neural network models for prediction, control, and optimization tasks in spray drying process [296,297]. MATLAB is widely used for solving complex mathematical models and simulating drying kinetics, especially in 1–2D domain of the spray drying process. It has extensive built-in functions and is extremely useful for parameter optimization and data processing from CFD simulations [212,298]. The key elements of both platforms are discussed in Table 8.

The major challenge of industrial scalability of the spray drying process is designing and controlling the operating parameters, such as temperature, air flow, for achieving enhanced performance and quality of the final product. To overcome these limitations, modeling tools such as ML have emerged that can accurately predict complex relationships and nonlinear interactions between variables by training patterns. In the spray drying process, ML models provide crucial information about the transport phenomena through a porous material with certain features. Therefore, the implementation of AI in the industrial sector has made it possible to obtain products of the highest quality and reduced manufacturing costs throughout the entire product value chain [301]. In a milk spray drying process plant, the ML approach was utilized for the prediction and fault detection of important performance parameters. A neural network-based NARX model was employed in the prediction of cyclone exit air temperature, which is considered as a key performance parameter that affects thermal efficiency. The model accuracy for fault finding was reported as 99.83% [290].

**Figure 12:**
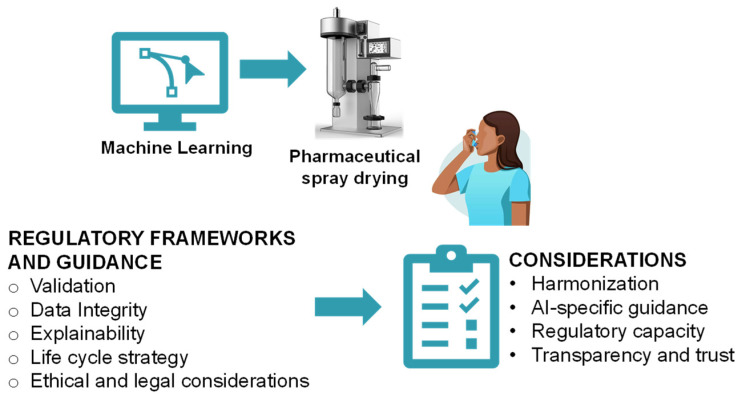
Regulatory frameworks for the implementation of ML in pharmaceutical spray drying [304].

ML technologies have been transforming various sectors, particularly the pharmaceutical industry. In pharmaceutical spray drying, ML can optimize batch production, allow predictive maintenance, enhance process control, and assist real-time quality monitoring [302]. Despite these benefits, regulatory agencies are aware of the unique threats of ML algorithms, such as continuous learning and uncertainty of the decision-making process, which pose a huge threat related to control, reproducibility, and traceability of the spray drying process [303]. This has tempted regulatory agencies to develop detailed frameworks and guidance (see Figure 12) for ML technologies that balance innovation, product quality, and patient adherence. Key regulatory challenges for implementing ML in the spray drying process include model validation and verification, data integrity, explainability and transparency, change management and lifecycle control, and ethical and legal considerations [304].

Model behavior may change over time. Therefore, regulatory authorities have advised a locked model during the time of validation, with a predetermined change control plan for any updates [305]. Data available must be reliable, accurate, and consistent for ML systems to utilize during training, testing, and deployment phases [306]. Moreover, the regulators demand that manufacturers understand the rational concept behind ML predictions and provide a clear justification based on scientific and engineering principles [307]. Further, regulatory authorities expect producers to define a model life cycle strategy that contains performance monitoring, retraining schedules, version control, and revalidation triggers. ML systems may accidentally introduce biases, particularly when trained on non-representative datasets. This may result in variable product quality during manufacturing. As a result, the regulatory authorities highlight the need for bias detection, fairness evaluation, and mitigation strategies as part of the model validation package [308].

The Food and Drug Administration (FDA) and European Medicines Agency (EMA) are the regulatory authorities that have been actively developing and releasing technical guidance and regulatory frameworks to address the incorporation of AI/ML in pharmaceutical drug delivery. A summary of key FDA/EMA elements is presented in Table 9.

Regulatory frameworks are not only about legal compliance, but they also promote trust, transparency, and fairness in AI systems. Explainability is considered crucial for promoting trust among stakeholders and clinicians, preventing algorithmic bias, and assuring that decisions are justifiable [314,315]. Therefore, explainable AI (XAI) is essential for transparency and explainability of ML models to the people, particularly in regulated domains such as the pharmaceutical industry. Regulatory authorities like the EU’s General Data Protection Regulation (GDPR) mandate that people affected by automated decisions have the right to get reasonable explanations about the ML models used in spray drying processes for drug manufacturing [316,317]. XAI helps users understand and trust model results by offering insights into the decision-making process, which is essential for regulatory authorities to evaluate the accuracy and integrity of ML-driven spray drying models [316,318]. The implementation of XAI promotes trust in AI systems by revealing how predictions are made, explaining relationships between input parameters and response variables, and quantitatively assessing the influence of the process parameters on predictions. This transparency is crucial for regulatory approval, as it facilitates ethical implementation and simplifies adherence to legal obligations [319,320]. In pharmaceutical drug manufacturing, explainable models can verify that decisions are made based on relevant trends, identify areas for improvement, and offer new perspectives on the data, all of which bolster regulatory review and approval [318,319].

### 5.2. Hybrid ML Models

The complexity of CFD simulation of the spray drying process increases with the level of physical details required, i.e., droplet atomization process, evaporation of single droplets, and modeling of the spray dryer unit. In the hybrid ML modeling approach, machine learning models are coupled with mechanistic-based model simulations. This integration allows benefiting from the advantages of both methods and eliminating their application shortcomings [321]. Hybrid ML models are commonly used to determine the optimal design of fundamental processes [322]. It also has applications in spray drying processes [323]. Hybrid ML models have been used for predicting the drug concentration in the spray drying process [324,325].

**Figure 13:**
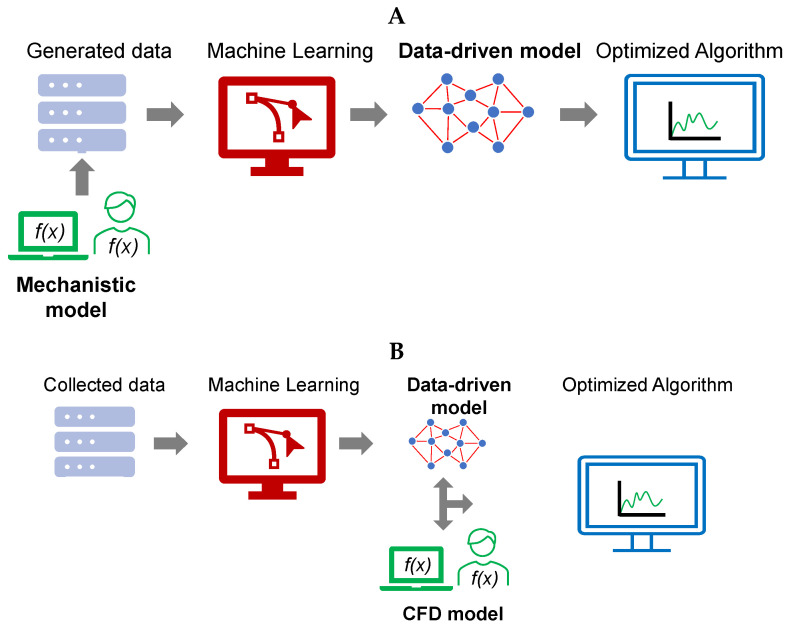
Schematic representation of a hybrid CFD-ML model: (**A**) CFD-based generated datasets approach; (**B**) ML model surrogate approach [333].

Combining ML and CFD models can be achieved via several approaches, as illustrated in Figure 13A CFD model is used to generate datasets for simulating the behavior of the physical phenomena under different experimental conditions (input parameters). This dataset is used by an ML algorithm to generate a trained ML model that can be used for prediction. In the second approach, as depicted in Figure 13B, the ML model is used as an input model (surrogate model) to the CFD model. The ML model is trained on data from physical experiments, simulations, or a combination of both. Therefore, it minimizes the need for repeated physical experiments. This approach was proposed to develop/optimize the spray drying process, thereby minimizing the requirement for costly experiments. Surrogate models are approximate mathematical models that closely mimic the behavior of a simulation model and are computationally efficient to evaluate. In a hybrid ML model, the data-driven model explains the system behavior based solely on data correlations [326], and the physical model provides a simplified representation of the real system that can be used to study, design, and test engineering problems. This hybrid approach helps in the development of an optimized algorithm by integrating the benefits of both physical and data-driven models. ML can help optimize parameters within the physical model, and the physical model can restrict the ML algorithm to prevent overfitting and ensure reasonable outcomes [327,328,329]. Therefore, by combining the two models, the hybrid ML model improves performance and reduces data requirements. It can also enhance prediction accuracy and decrease training complexity compared with single-algorithm approaches [330]. CFD combined with a mechanistic model was developed for a better understanding of the spray drying process of sticky materials. CFD model optimized the nozzle conditions by generating a large dataset of droplet size distribution for a subset of nozzles and spray angles. This data was directly fed into the mechanistic model for the prediction of heat and mass transfer between the gas and droplet, outlet temperature, size separation, residual solvent content in the particle, and droplet drying time [331]. The droplet drying kinetics of Lonicerae Japonicae Flos (LJF) extract were simulated using a CFD model to calculate the moisture removal rate of atomized droplets in the drying chamber. The CFD model was verified using experimental temperature field measurements. The experimental and simulation data were fed into a deep reinforcement machine learning algorithm for optimizing the spray drying process [332].

**Figure 14:**
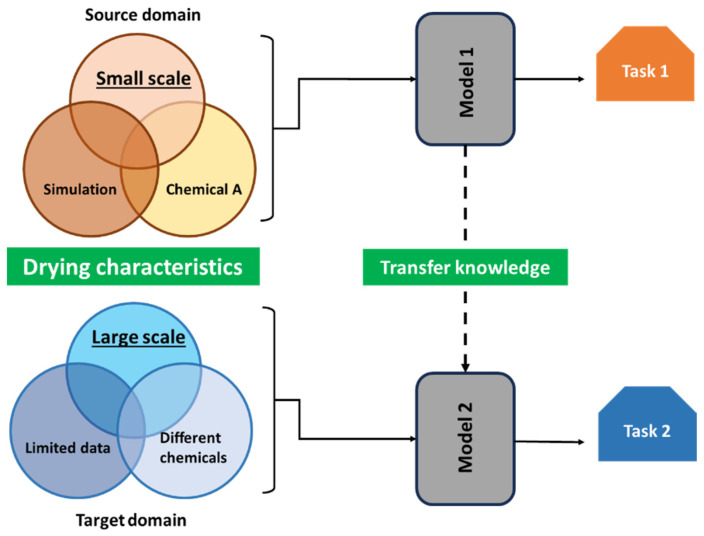
Digital twin approach for predicting energy efficiency respectively [337], reproduced with permission from Elsevier, 2025.

Energy efficiency is vital in the spray drying process as it can significantly improve the product yield, energy utilization, and minimize the operational costs. However, due to limited data issues, analyzing the performance of energy efficiency using an ML-based model is ineffective and inaccurate [334] To overcome these drawbacks, digital twin technology has recently emerged as a powerful tool for real-time process monitoring and decision-making by integrating with physical machines [335,336]. Digital twin approaches support the ML model by initiating parameters using limited simulated data from the source domain for effective prediction of energy efficiency, as shown in Figure 14. The digital twin-aided transfer learning model was verified in an industrial detergent powder spray drying process. Results suggest that the proposed model enhanced the drying efficiency by 14.53% and reduced the additional energy on demand and supply by 50.05% and 81.27%, respectively [337]. The digital twin approach delivers a comprehensive and real-time predictive view of process-equipment performance and prospective behavior, enabling early fault detection, remote troubleshooting, and improved operational reliability [338,339]. It can help in detecting possible defects, enabling remote troubleshooting, and eventually enhance customer satisfaction. Further, it helps with product distinction, product quality, and other supplementary services. The digital twin technique processes huge amounts of sensitive data, raising significant privacy concerns. Unauthorized access or data breaches can lead to operational interruptions and sacrifice critical infrastructure [340]. Lack of integrated management and decision-making strategies, along with partial monitoring and modeling, can leave digital twin systems exposed to cyber-attacks [341]. Adopting end-to-end encryption, regular security audits, and proactive threat detection mechanisms are crucial for protecting digital twin environments [342]. Real-time monitoring and strong asset authentication protocols are essential to maintain data integrity and respond quickly to potential breaches [339]. The effectiveness of digital twins depends largely on the quality and accuracy of the data they receive. Inconsistent data from sensors or other sources can sacrifice the reliability of the digital twin, leading to misleading insights and decision-making. Ensuring the authenticity of the data, particularly from the operational section, is essential for preserving the data integrity [341].

### 5.3. Limitations of Using ML/AI in Engineering

ML and AI have several advantages in engineering, including enhanced efficiency, improved decision-making, and advanced automation [241]. However, there are several limitations to combining ML and AI models in engineering systems. The key limitations include testing, AI software quality, and data management [343]. Other limitations include data quality issues, design method challenges, and performance concerns related to the ML model during the production stage [344]. AI and ML models largely depend on high-quality data to train the appropriate models; however, such data can be limited or difficult to obtain in complex system engineering [345]. Poor data quality can result in inaccurate predictions and decisions. Previous experimental data may also be limited, making it difficult to develop and train appropriate models [346]. Furthermore, the data may include historical biases, cultural stereotypes, or unequal representations of various groups, resulting in biased or discriminatory outcomes [347]. Biased algorithms can produce designs that do not effectively reflect the requirements of all users [348]. Combining AI/ML technologies with existing systems and software can be demanding, owing to compatibility issues resulting from differences in data formats, communication protocols, or architectural styles [346].

### 5.4. Comparative Analysis Between CFD and ML Models

CFD modeling provides detailed information about complex interactions between chemical and physical properties, fluid dynamics, and heat or mass transfer that occur during the spray drying process. It can simulate the droplet drying kinetics and their influence on powder properties, which is vital for pharmaceutical drug delivery [349]. Further, CFD models provide useful theoretical information for knowing the fluid flow patterns, droplet trajectory, and evaporation mechanism inside the spray drying tower. Advanced CFD-DEM models consider both fluid and particle interactions, which is significant for a spray drying process [350]. However, due to the complexity of the spray drying process, computational powder may be insufficient for industrial scalability. Most of the CFD models are focused on resolving fluid dynamics rather than on the final product output properties, also, experimental validation of CFD models is often limited [351].

ML models have been used to boost analytical models. In a recent study, the prediction accuracy of an ML model was compared with a simplified CFD model. The prediction accuracy of the ML model was six times better than that of the CFD model. Therefore, ML models can enhance the prediction accuracy, minimize the computational cost, and thereby speed up the process development. However, an ML model requires huge experimental datasets to make accurate predictions. Moreover, ML models are still an emerging technique, as most of the pharmaceutical spray drying applications have used ML models to assist the existing models and not fully replace them [352]. A comparative analysis of the CFD model and ML model in terms of different features is shown in Table 10.

A hybrid CFD-ML model combines the strengths of both the approaches and helps in making more accurate predictions than traditional CFD alone. However, ML models require reference data for training, emphasizing the need for advanced hybrid methods that enhance predictive power while reducing resource requirements Transfer learning is a well-established technique for mitigating data scarcity [353,354,355]. This approach adapts the knowledge gained from training a model on the source task to enhance performance on a different but related task (the target task). Instead of training a model from scratch for each new task, transfer learning adapts a pre-trained model to a new, often more specific, task. Further, this technique is particularly useful when the target task has limited data, as it involves reusing learned features, representations, or parameters from the source model to optimize the target model, leading to faster convergence and reduced computational cost and time.

**Table 10:** Summary Comparison between CFD and ML model.

Characteristics	CFD Model	ML Model	Refs.
Mechanistic insight	Simulates physical phenomena, droplet drying kinetics, and process robustness	Depends on data patterns and has less physical insight	[350,352]
Prediction accuracy	Good: Based on model validation and computational resources	Higher with substantial datasets	[349,351,352]
Computational expense	High: Particularly for industrial scale	Low: predictions are rapid upon training	[351,352]
Product quality prediction	Able to model the influence of operating parameters on powder properties	Able to enhance the prediction accuracy, but less mechanistic	[349,352,356]
Industrial Applicability	Limited due to scale and validation	Potential for automation and quick process optimization	[351,352]

## 6. Conclusions

This review discusses the past and current state of research on modeling the spray drying process, particularly in the pharmaceutical industry. Spray drying is widely used in the food, chemical, and pharmaceutical industries. In the pharmaceutical industry, it is used for drug delivery via oral, nasal, transdermal, and inhalation routes. The spray-drying process involves heat and mass transfer, which results in the drying of droplets, followed by the formation of dry particles. Process parameters such as the inlet temperature, outlet temperature, drying gas flow rate, spray gas flow rate, feed concentration, and feed flow rate play a crucial role in determining the properties of the final dry powder. CFD modeling is commonly used in the spray-drying process for modeling the dryer unit. However, these models have many drawbacks, such as longer computational times, difficulty in experimental validation and determining drying kinetics, and inability to predict complex spray dryer conditions without the help of sub-models. ML models can be used to accurately predict spray drying process parameters and CQAs of the product. In this review study, we highlight recommendations concerning the challenges of CFD modeling in the spray drying process, which include the use of machine learning-based models, hybrid models, efficient computing methods, and validation practices. Coupling CFD models with novel modeling techniques, such as the reaction engineering approach and the drying kinetics model for enhancing model accuracy, using an advanced mesh refinement (AMR) strategy to perform cost-effective simulations, combining CFD models with DEM for determining the operating parameters, and utilizing a multi-scale approach to model the transport phenomena in a spray drying process. Advancements have been made in the application of ML models to the spray-drying process to overcome the limitations of CFD-based modelling. Digital twin-aided transfer learning is a recently emerged technique that assists ML models in making effective predictions using limited data. DoE datasets were also combined with ML models to improve prediction accuracy. Implementation of ML in the industrial sector is also possible through proper consideration of the regulatory framework. The FDA/EMA has released technical guidelines to address the implementation of AI/ML in the pharmaceutical sector. Explainable AI (XAI) is mandated by regulatory authorities to ensure the transparency and explainability of ML models to protect people affected by automated decisions. Nevertheless, ML models also have a few limitations, such as requiring high-quality datasets for better predictions, which are ineffective in predicting the influence of new process parameters on product quality. Moreover, this review outlines the lack of research utilizing the ML approach in spray drying, especially in the pharmaceutical field. Obtaining high-quality data consistently and using more advanced systems and software could improve the ML model prediction accuracy in the spray drying process. The utilization of the ML approach in the spray-drying process cannot be successful without proper experimental data to explain the CQAs of the final dried powder.

## Figures and Tables

**Table 1:** Summary of different drying methods and their limitations.

Drying Technology	Process Description	Drawbacks	Refs.
Freeze drying	Products are frozen and subjected to a vacuum to maintain product quality	High processing time (24–48 h) High production costs and energy consumption	[12,13,14,15]
Fluidized bed drying	Particles are suspended and mixed in a hot air stream for constant and efficient drying	Low product quality Drying temperature limitations	[16,17,18,19]
Spray–freeze drying	The product is sprayed, then frozen, and finally dried under vacuum	Time-consuming (3 steps) Complex Expensive Fragile particles	[20,21]
Electro spraying	Liquids are sprayed into fine droplets using an electric field, and dried by evaporation	Reduced production efficiency High cost	[22,23]
Solar drying	Direct sunlight is used for drying products	Climate dependent Additional heat and large area requirement Non-uniform drying	[24,25,26,27]
Superheated-steam drying	Drying of products by heating the steam above its boiling point	Unwanted color changes in products Temporary moisture increase	[28,29,30,31,32]
Infrared drying	Drying of products takes place using thermal radiation	Weak penetrative ability Product overheating and burning	[33,34,35,36]
Supercritical fluid drying	Supercritical fluids, such as CO_2_, are used to remove the water from the product	High cost Requires organic solvent to improve water solubility and drying efficiency	[37,38,39,40,41,42]

**Table 2:** Summary of studies applying the DoE approach in pharmaceutical spray drying.

Design Methodology	API & Excipient	Critical Process Parameters	Critical Quality Attributes	Refs.
**Response Surface Methodology**	Human type 5 adenoviral vector vaccine, mannitol/dextran	Inlet temperature, feed flow rate, and feed concentration	Residual moisture content and process yield	[160,161]
**2^3^ factorial design**	Ivermectin, L-leucine	Inlet temperature, feed rate, and atomization air flow rate	Powder yield, particle size, and morphology	[162,163,164,165]
**Box–Behnken design**	Resveratrol, low-methoxyl pectin (LMP), and caprylic/capric glycerides (CCG)	Feed rate, inlet temperature, Solute concentration (%*w*/*v*), outlet temperature, and solvent concentration (%*v*/*v*)	Moisture content, particle size, powder yield, and particle size distribution	[165,166,167,168,169]
**2^4^ full-factorial design**	Disodium cromoglycate, mannitol	Feed rate, feed concentration, inlet temperature, and drying gas flow rate	Particle size distribution, powder yield, residual solvent content, and outlet temperature.	[170,171]
**Half-factorial design**	Bacteriophage MS2 VLP-based candidate vaccine, mannitol, l-leucine, trehalose, and dextran	Effect of excipient ratio, feed rate, and atomization pressure	Particle size, moisture content, yield	[172,173]
**Central composite face-centered design (CCFD)**	Fenofibrate, mannitol, and trehalose	Ratio of two carriers, crystallinity of spray-dried powder, and solvent ratio	Particle size, batch yield, and antioxidant and antimicrobial activity	[174,175]
**Circumscribed central composite design**	Diazepam, mannitol	Water/organic solvent ratio, liquid feed flow rate, total solid content, atomizing air flow rate, and type of organic solvent	Dissolution rate, yield, actual drug load, particle size, and crystallinity of drug and excipient	[176]
**3 × 4 full factorial design**	Cationic liposomal adjuvant formulation 01 (CAF01), lactose, mannitol, and trehalose	Choice of stabilizing excipient and the lipid concentration	Yield, moisture content, polydispersity index, particle size, and particle morphology	[177]
**Factorial 2 × 2 × 3 experimental design**	Enhanced green fluorescent protein (EGFP) and luciferace (FLuc) Dicer substrate asymmetric duplex siRNAs, trehalose, lactose, and mannitol	Excipient concentration and the ratio of nanoparticle to excipient	Moisture content, particle morphology, particle size, and powder yield	[178]

**Table 3:** Summary of recent research studies using CFD tools in various spray drying applications.

Product	Application	Research Aim	Process Parameters	Main Findings	Refs.
Tiotropium bromide nanoliposomes (trojan)	Pharmaceutical	To produce Tiotropium bromide nanoliposomal dry powder for inhalation using the thin-film hydration and spray drying method	Inlet temperature (°C)—110 ± 5 Aspiration capacity (%)—85 Feed rate (mL/min)—10	Newly produced Trojan dry powder showed great promise for the treatment of respiratory diseases CFD analysis showed that higher inhalation flows increase particle deposition in airways due to greater inertia and turbulence	[199]
Fresh whey	Food	To fill the gap in the literature between fluid dynamics and spray drying of fresh whey, and provide a detailed assessment of important process parameters and design properties	Inlet temperature (K)—413.15; 453.15; 493.15; 513.15; 573.15	The lowest drying temperature is the optimal process condition, as it resulted in the lowest particle deposition and the highest thermal efficiency Parameters such as air flow rate and nozzle need to be further explored to understand their influence on thermal efficiency and powder recovery	[200]
Nanostructured silica particle	Chemical	To properly evaluate the spray drying process, with a specific focus on nanostructured silica particle formation from sodium silicate precursor	Inlet temperature (K)—473, 673, and 873 Atomization flow rate (L/min)—2, 4, and 6	CFD simulations properly established the evaporation rate constant (K) and the temperature difference (ΔT) inside the droplets.	[201]
Mold powder slurry	Chemical	To explain mold powder spray drying using a mathematical model that describes droplet and granule movement as well as heat and mass transfer, and explore the influence of process parameters on granule size using CFD software	Inlet temperature (K)—673–1073 Atomization pressure (MPa)—1.5–2.3 Slurry mass flow rate (kg s^−1^)—0.01–0.1	Effect of spray drying process parameters such as inlet temperature, atomization pressure, and slurry mass flow rate on the final granule was studied using the CFD model, and results suggested that slurry mass flow rate largely influenced the final granule size	[202]
Guava juice formulation	Food	To model the spray-drying of a formulation of guava juice using a CFD approach	Mass flow rate (kg/h)—81 Inlet temperature (K)—453	Simulation results suggest the need to implement drying kinetics with experimental support, and suitable inlet flow conditions to perform detailed CFD simulations of the spray-drying of fruit juices	[203]
SiO_2_ and ZnO particles	Chemical	To study the modelling of ZnO-SiO_2_ Composite through a consecutive electrospray and spray drying method	Atomization flow rate (L/min)—2–10	Applied voltage and the precursor flow rate effectively affected the composite droplet size, whereas gas flow rate and inlet temperature influenced the effectiveness of the composite particle formation in the spray drying process.	[204]
Lime slurry	Food	To investigate the applicability of three drying models and three turbulence models in analyzing the drying process in a laboratory spray dry scrubber	Inlet temperature (°C)—108.8; 130.3; 142.5 Mass flow rate (kg/s)—1.1	Hindered drying mechanistic model is superior to the commonly used d^2^ law and perfect shrinkage models, and therefore should be preferable in drying applications	[205]
L-leucine	Pharmaceutical	To develop a new CFD-based model of complex transport and droplet drying kinetics in a lab-scale spray dryer, and relate CFD-predicted drying parameters to powder aerosolization performance from a reference dry powder inhaler (DPI)	Liquid feed rate (%)—100 Gas flow rate (L/min)—120	Reducing the CFD-predicted maximum drying rate experienced by droplets improved the aerosolization performance of the powders aerosolized with a reference DPI	[206]
Skim milk powder	Food	To simulate the particle movement within the spray dryer, and account for the observed stickiness of the skimmed milk powder	Atomization flow rate (kg/s)—0.0139 Inlet temperature (°C)—195	CFD simulations provided accurate predictions of the activity inside the spray dryer, and they agreed with the experimental results Stickiness arises during the spray drying process, and it can be utilized to control particle agglomeration to obtain better quality powder	[207]

**Table 4:** Characteristics of ANSYS Fluent and OpenFOAM platforms.

Characteristics	ANSYS Fluent	OpenFOAM	Refs.
Computational cost	High	Low, but may compromise accuracy	[216,217]
Accuracy	High	Comparable	[218,219]
Usability	User-friendly, detailed guides and tutorials	Limited, harder learning curve for new users	[220,221]
Numerical methods	Finite-Volume-Method (FVM) and electromagnetic equations with Finite-Difference-Method (FDM)	Finite-Volume-Method (FVM) for all equations	[222,223]
Multiphase modeling	Geo-reconstruct scheme, implicit approach	Different approaches to interface compression, explicit solutions	[224,225]
Customization	User-defined functions	Highly customizable, enables integration of latest numerical methods	[217,220]
Computational resources	High computational resources and knowledge required	Less resource-intensive	[217,226]

**Table 6:** Comparison of prediction accuracies of different ML models in the spray drying process.

Research aim	ML Algorithm	Dataset	Prediction Accuracy	Refs.
Physical stability of solid dispersions at 3 months and 6 months	Random forest (RF), LightGBM, Support vector regression (SVR)	Fifty drug compounds with ten molecular descriptors	RF—82.5% (highest) LightGBM—80.83% SVM—77.50%	[288]
Prediction of median spray-dried dispersion particle size (SDD)	Partial least square (PLS), Support vector regression, Neural networks (multi-layer perceptron)	680 SDD lots using 57 different pressure nozzles across two spray dryer scales and 88 unique APIs	PLS—7.69 and 6.81 (training and testing RMSE) SVR—5.39 and 6.56 Neural network—3.72 and 6.10	[131]
To obtain an effective solid dispersion formulation design for the oral administration of water-insoluble drugs	RF, SVR, LightGBM	Three data sets–physical stability (646 lots), dissolution curves (702 lots), dissolution profiles (4214 samples)	RF—77.7% LightGBM—76.4% SVR—59.7%	[289]
Prediction and fault detection in key performance parameters for a milk spray drying process plant	Decision tree, random forest, logistic regression, and SVM	17400-time instances	Decision tree—99.85% Logistic regression—99.59% Random forest—99.85% SVM—95.40%	[290]

**Table 7:** Summary of the combination of DoE and ML models used in the spray drying process.

DoE	ML Model	Product	Process Parameters	Response Variables	Prediction Accuracy	Refs.
Response surface methodology (RSM)	Artificial Neural Network (ANN)	Aripiprazole -cyclodextrin complex	Feed rate, inlet temperature, feed concentration, compressed air flow rate, and aspirator capacity	Powder yield, moisture content	R^2^ = 0.854 for yield, R^2^ = 0.886 for moisture content	[293]
2^4^ full-factorial design	Extreme gradient boosting	α-lactose monohydrate	Feed rate, outlet temperature (T_out_), and solid concentration.	Powder yield, residual moisture content, cut off diameter (X_50_)	R^2^ = 0.982 for yield, R^2^ = 0.998 for residual moisture content, R^2^ = 0.923 for X_50_	[294]
Quality by design (QbD)	Random Forest	Deionized water	Aspirator rate, inlet temperature	Outlet temperature (Tout)	R^2^ = 0.99	[295]
DoE–4 factors (two continuous and two categorical factors)	Support vector machine (SVM) and ANN	Lactose/Polyvinylpyrrolidone and lactose/Kollidon physical mixtures	Type of core and shell materials and their concentrations	Powder compactibility	Root mean square error (RMSE) = 2.3% for ANN, RMSE = 6.8% for SVM	[292]

**Table 8:** Key elements of TensorFlow and MATLAB machine learning platforms.

Features	TensorFlow	MATLAB	Refs.
Application	Trains advanced neural networks for accurate predictions and process optimization in spray drying process	Mathematical modeling, simulation of drying kinetics, and experimental data analysis	[217,298]
Flexibility	Highly flexible	Less flexible and inefficient in handling advanced neural networks	[296,297]
Scalability	Highly scalable for large and complex datasets	Limited due to model dimensionality	[296,297]
Experimental analysis	Not generally used for direct experimental validation	Effective for image processing and data analysis	[299,300]
Computational efficiency	Suitable for large-scale complex computations	Less suitable for complex system modeling	[297,299]

**Table 9:** Key FDA/EMA aspects in pharmaceutical drug manufacturing.

Element	FDA	EMA	Refs.
AI/ML in drug manufacturing	Discussion paper, lifecycle management, SaMD framework	AI work plan till 2028, valid AI algorithms for data analysis in Pharmacopeia	[309]
Algorithm categories	Locked vs. adaptive/continuous learning	Endorsement of AI for data evaluation	[309]
Main emphasis areas	Bias mitigation, transparency, real-world monitoring	Responsible utilization, risk management, training	[310]
International Collaboration	Joint good practice principles with Health Canada and the UK’s MHRA	Partnership with HMA (Head of Medicines Agencies), open access to methodologies	[311,312]
Regulatory status	Progressing, with explicit guidance expected	Ongoing, with published work plans and guidelines	[313]

## Data Availability

Not applicable.

## Abbreviations

AMR	Advanced Mesh Refinement
ANN	Artificial Neural Network
API	Active pharmaceutical ingredient
CFD	Computational Fluid Dynamics
CPP	Critical process parameters
CQA	Critical quality attributes
DoE	Design of Experiment
DNN	Deep neural network
DPI	Dry powder inhaler
EMA	European Medicines Agency
FDA	Food and Drug Administration
GBR	Gradient Boosting Regression
GDPR	General Data Protection Regulation
ML	Machine Learning
Pe	Peclet number
QBD	Quality by Design
REA	Reaction Engineering Approach
RMSE	Root mean square error
RSM	Response Surface Methodology
RTD	Residence time distribution
SVM	Support Vector Machine
XAI	Explainable AI
